# Transcriptomic features of immune inflammation and neural plasticity associated with early neurological improvement in acute ischemic stroke patients with large vessel occlusion

**DOI:** 10.3389/fnins.2025.1581758

**Published:** 2025-08-14

**Authors:** Dong Zhang, Xinying Jing, Ruinan Ma, Zhizhang Li, Xiaoguang Zhang, Ying Ding, Yunhua Yue

**Affiliations:** Department of Neurology, Yangpu Hospital, School of Medicine, Tongji University, Shanghai, China

**Keywords:** acute ischemic stroke, large vessel occlusion, early neurological improvement, transcriptomics, immune-inflammatory response

## Abstract

**Background:**

Acute ischemic stroke (AIS) caused by large vessel occlusion (LVO) is a leading cause of disability and mortality worldwide. Although endovascular thrombectomy (EVT) has significantly improved outcomes, many patients do not achieve early neurological improvement (ENI) despite timely reperfusion. This study aims to investigate the peripheral blood mRNA molecular characteristics and underlying mechanisms associated with ENI after EVT in AIS-LVO patients, to inform individualized treatment and optimize prognosis.

**Methods:**

This retrospective study included AIS-LVO patients who underwent EVT between January 2022 and December 2023. Peripheral blood samples were collected before reperfusion therapy for mRNA transcriptome sequencing and bioinformatic analysis. Patients were grouped according to ENI status. Differentially expressed genes (DEGs) were identified, and functional enrichment, signaling pathway, and protein-protein interaction network analyses were performed.

**Results:**

A total of 108 patients were initially screened, and 20 were ultimately included in the study (13 in the ENI group and 7 in the non-ENI group). Among the 20,501 genes detected, 752 were found to be significantly differentially expressed, with 208 upregulated and 544 downregulated. The upregulated genes in the ENI group were mainly enriched in immune and inflammatory response pathways, such as NOD-like receptor and Toll-like receptor signaling. In contrast, the downregulated genes were primarily associated with neurodevelopment and synaptic plasticity. Protein-protein interaction analysis identified immune-related genes, including JUN, IL1A, and CCR5, as central nodes. Overall, the ENI group exhibited a dynamic balance between immune activation and suppression of neuronal plasticity.

**Conclusion:**

This study reveals the transcriptomic characteristics of ENI in AIS-LVO patients during the acute phase, highlighting the protective roles of moderate immune-inflammatory activation and suppression of neural plasticity in acute injury response. The identification of related molecular pathways and biomarkers provides a theoretical basis for individualized treatment and improved functional outcomes. Future studies should expand sample size and integrate single-cell sequencing and liquid biopsy multi-omics approaches to further clarify the molecular mechanisms of ENI and promote clinical translation.

## Introduction

Acute ischemic stroke (AIS), particularly large vessel occlusion (LVO), remains one of the leading causes of global disability and mortality. Endovascular thrombectomy (EVT) has become a groundbreaking therapy for AIS, successfully restoring blood flow in more than 70% of patients and greatly enhancing functional recovery during the acute stroke phase (Goyal, 2016; Albers et al., 2019). Despite the timely administration of reperfusion therapy, a significant number of patients do not exhibit early neurological improvement (ENI), which is defined by a swift reduction in neurological deficits following EVT (Saver et al., 2016). This underscores the necessity of identifying molecular predictors of ENI to inform personalized therapeutic strategies and enhance post-intervention care.

Recent advancements in high-throughput omics technologies, particularly in transcriptomics, have enabled innovative investigations into the pathophysiological mechanisms underlying AIS and its related complications. Previous transcriptomic studies have elucidated complex transcriptional responses to ischemia-reperfusion injury, revealing molecular pathways involved in neuroinflammation, oxidative stress, synaptic plasticity, and cellular survival mechanisms (Movahed et al., 2021). A study investigating the temporal changes in physiological and molecular markers across various brain regions following transient global ischemia in rat models emphasized the roles of inflammation, oxidative stress, and apoptosis in ischemic injury. It highlighted the critical functions of brain-derived neurotrophic factor (BDNF) and pro-inflammatory cytokines in post-stroke recovery, suggesting that these factors are central to the recovery process (Kapoor et al., 2019). Furthermore, the transcriptional activation of anti-apoptotic genes, such as BCL2, alongside the suppression of pro-inflammatory cytokines like IL6, has been associated with neuroprotection and improved recovery in experimental stroke models (Anrather, 2016; Jayaraj et al., 2019). In a clinical context, blood-based mRNA biomarkers, including glial fibrillary acidic protein (GFAP) and neurogranin (NRGN), have shown potential in predicting stroke severity and functional outcomes (Bustamante et al., 2017). These studies collectively underscore the importance of mRNA profiling in elucidating the complex molecular responses to ischemic brain injury and highlight potential pathways for therapeutic intervention. By understanding the gene expression changes that occur during and after ischemic events, researchers can identify new targets for drug development and improve treatment strategies for stroke patients. However, transcriptomic analyses related to early neurological improvement in patients with large vessel occlusion treated with thrombectomy are still lacking.

This study aims to explore the key molecular pathways and potential molecular biomarkers associated with early neurological improvement. Identification of these biomarkers will help deepen the understanding of the biological mechanisms underlying recovery after thrombectomy and provide a theoretical basis for developing individualized treatment strategies and improving functional outcomes.

## Materials and methods

### Patient selection and sample collection

This study retrospectively analyzed a cohort of large vessel occlusive stroke patients who had undergone EVT, with data collected prospectively at Yangpu Hospital, Tongji University School of Medicine, from January 2022 to December 2023. To ensure the reliability of the results, we screened patients and excluded those without available blood samples, as well as those whose conditions could affect the study analysis or transcriptomic profiling. The exclusion criteria included: (1) a history of malignancy; (2) participation in other drug-related studies; (3) failure to meet RNA quality control standards; and (4) lack of essential clinical laboratory test results.

We collected blood samples from all patients prior to reperfusion treatment, and these samples were stored in a −80°C freezer for subsequent experimental analysis.

The study received approval from the Institutional Review Board at Yangpu Hospital, affiliated with Tongji University School of Medicine (LL-2021-LW-002, February 22, 2021). Every clinical investigation was carried out in accordance with the Declaration of Helsinki's principles. The retrospective design of the study meant patient consent could not be secured, but it was waived, and patient data confidentiality was protected at Yangpu Hospital of Tongji University School of Medicine.

### Clinical variable assessment

Patient records, encompassing admission data, were retrospectively analyzed to evaluate demographics, vascular risk factors, imaging, and laboratory data. Two certified neurologist employed the National Institutes of Health Stroke Scale (NIHSS) to assess neurological deficits (Sucharew et al., 2013). The ENI NIHSS definition was documented for patients at the start and again after 24 h. ENI was characterized by a reduction in NIHSS of 8 or more, or a drop to 0 or 1, 24 h after the patient first arrived at the hospital (Heinze et al., 2023). The Alberta Stroke Program Early Computed Tomography Score (ASPECT) score from the Alberta Stroke Program was utilized to evaluate pre-treatment infarction volume via early Computed Tomography (CT) (Barber et al., 2000). The American Society of Interventional and Therapeutic Neuroradiology/Society of Interventional Radiology grading system was used to assess collateral circulation, Grades 0–1 represent insufficient collateral circulation, while grades 2–4 indicate moderate to excellent (Zaidat et al., 2013). Stroke etiology was classified based on the criteria of Trial of Org 10,172 in Acute Stroke Treatment (Adams et al., 1993). Successful recanalization was defined as modified thrombolysis in a cerebral infarction score of 2b or 3 (Pego-Pérez et al., 2019). The Heidelberg Bleeding Classification was used to diagnose symptomatic intracerebral hemorrhage (sICH) within 48 hours of EVT (Von Kummer et al., 2015).

### mRNA analyses

Samples had their total RNA extracted with Trizol Reagent (Invitrogen), and a NanoDrop spectrophotometer was used to assess its concentration, quality, and integrity. mRNA was enriched from total RNA using poly-T oligo-attached magnetic beads and fragmented in Illumina proprietary fragmentation buffer under elevated temperatures. First-strand cDNA synthesis was performed using random primers and SuperScript II, followed by second-strand synthesis using DNA Polymerase I and RNase H. The cDNA fragments were end-repaired, adenylated at the 3′ ends, and ligated with Illumina sequencing adapters. Fragments of 400–500 bp were selected using the AMPure XP system (Beckman Coulter), amplified by PCR for 15 cycles, and quantified using an Agilent Bioanalyzer 2100. The prepared libraries were sequenced on a NovaSeq 6000 platform (Illumina) at Genekinder Medicaltech (Shanghai), China.

### Bioinformatic analysis

Raw sequencing data were filtered using fastp (v0.22.0) to remove adapters and low-quality reads, generating clean data. The filtered reads were aligned to the reference genome with HISAT2 (v2.1.0), and gene expression levels were quantified as FPKM values using HTSeq (v0.9.1). Differentially expressed genes (DEGs) were identified using DESeq2 (v1.38.3) with thresholds of |log2FoldChange(FC)| ≥ 1 and a Benjamini–Hochberg adjusted *p*-value (FDR) < 0.05 to control for multiple testing. Hierarchical clustering and visualization of DEGs were performed using the pheatmap package in R.

To explore the biological functions of DEGs, Gene Ontology (GO) and Kyoto Encyclopedia of Genes and Genomes (KEGG) pathway enrichment analyses were conducted using topGO (v2.50.0) and ClusterProfiler (v4.6.0), respectively. For both analyses, the Benjamini–Hochberg procedure was applied to adjust for multiple comparisons, and terms with adjusted *p*-values (FDR) < 0.05 were considered significantly enriched. Protein-protein interaction (PPI) networks of DEGs were constructed and visualized using the STRING database to identify potential regulatory relationships. Finally, gene set enrichment analysis (GSEA) was performed using GSEA software (v4.1.0).

### Statistical analysis

Statistical analyses were conducted utilizing SPSS version 26.0. Categorical data were compared using the chi-squared (χ^2^) test. For continuous data, the Kolmogorov-Smirnov test was employed to assess normality. Data conforming to a normal distribution were represented as mean ± standard deviation (x ± s) and analyzed using independent sample *t*-tests. Non-normally distributed data were presented as median (Q1–Q3) and analyzed using the Mann–Whitney *U*-test. Clinical covariates demonstrating statistical significance (*P* < 0.05) in either adjusted correlation or univariate analyses were considered.

## Results

### Features of patients' clinical profiles

**Figure 1:**
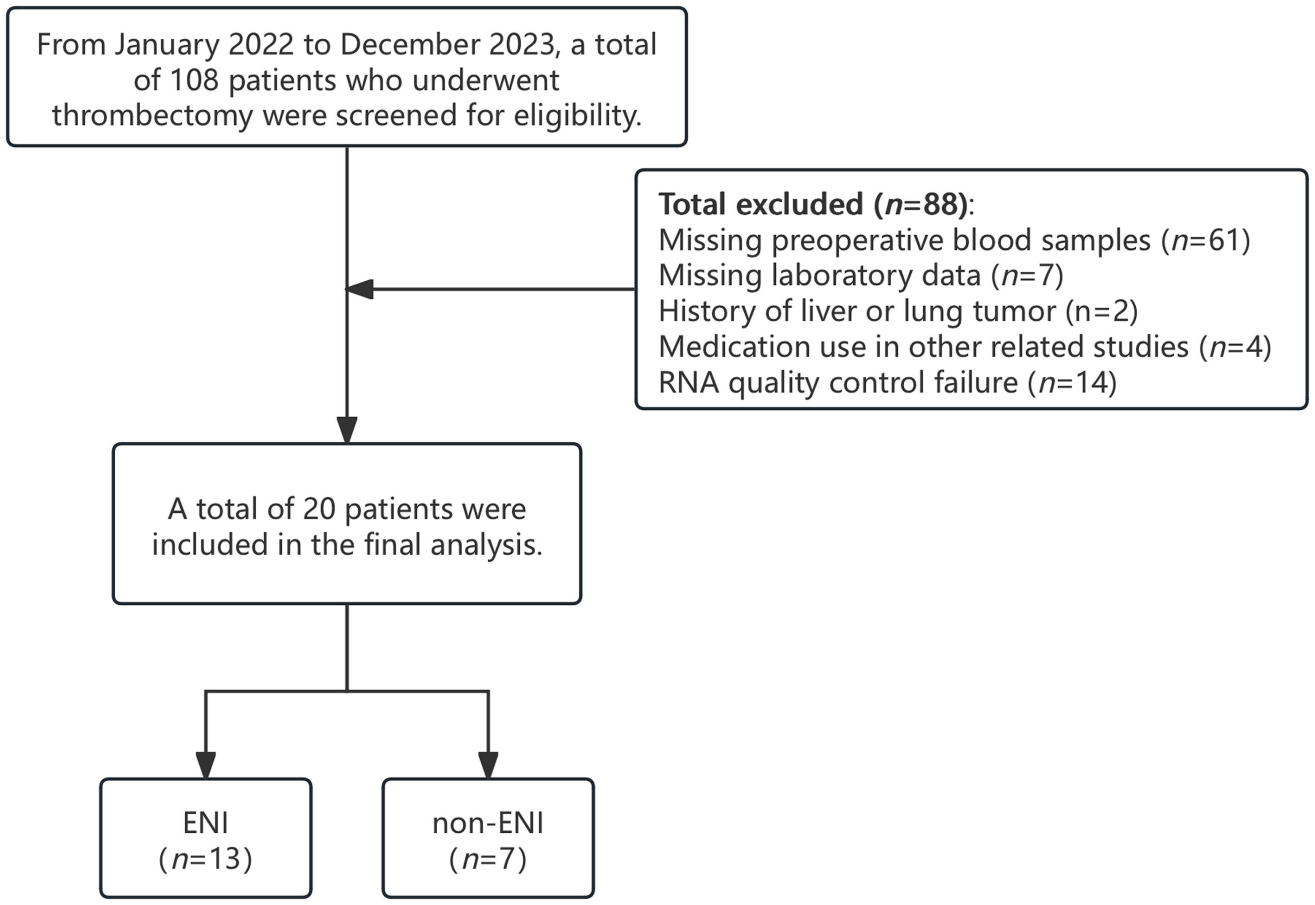
Research flow chart.

From January 1, 2022, to December 31, 2023, a total of 108 patients with AIS-LVO who underwent EVT at our center were screened for inclusion in this study. Among them, 88 patients were excluded due to missing preoperative blood samples, a history of malignancy, incomplete laboratory data, participation in other drug-related studies, or failure in RNA quality control. Ultimately, 20 patients were included in the final analysis, comprising 13 with ENI and 7 without ENI (Figure 1). All patients demonstrated effective collateral circulation. The median age was 77 years (63–89 years), and 45.0% were male. Regarding relevant medical history, 60.0% of patients had hypertension, 50.0% had diabetes mellitus, and 55.0% had coronary artery disease (CAD). At baseline, the median NIHSS score was 10, and the median ASPECTS prior to treatment was 8. Thirteen patients (65.0%) received intravenous thrombolysis before EVT. Table 1 summarizes the laboratory and clinical data in detail. There were no statistically significant differences in these baseline characteristics between the ENI and non-ENI groups.

**Table 1:** Baseline information for patients experiencing both ENI and non-ENI.

**Variables**	**All (*n* = 20)**	**ENI (*n* = 13)**	**non-ENI (*n* = 7)**	***P*-value**
Age (years), median (IQR)	77 (63–89)	75 (61–89)	84 (68–90)	0.606
Male, n (%)	9 (45.0)	7 (53.8)	2 (28.6)	0.374
**Risk factors**, ***n*** **(%)**				
Smoking	11 (55.0)	7 (53.8)	4 (57.1)	1.000
Diabetes	10 (50.0)	8 (61.5)	2 (28.6)	0.350
Stroke/TIA	4 (20.0)	2 (15.4)	2 (28.6)	0.587
Hypertension	12 (60.0)	8 (61.5)	4 (57.1)	1.000
Coronary artery disease	11 (55.0)	8 (61.5)	3 (42.9)	0.642
Atrial fibrillation	10 (50.0)	8 (61.5)	2 (28.6)	0.350
**Clinical data**				
Baseline NIHSS, median (IQR)	10 (5–20)	18 (6–21)	7 (6–9)	0.301
Baseline ASPECTS, median (IQR)	8 (5–10)	9 (5–10)	8 (4–10)	0.717
Systolic blood pressure (mmHg), median (IQR)	164.50 (141.25–170.00)	155.00 (133.50–173.50)	165.00 (164.00–170.00)	0.339
Diastolic blood pressure (mmHg), median (IQR)	86.50 (73.00–96.75)	82.00 (64.00–95.00)	88.00 (75.00–100.00)	0.383
Time from onset to recanalization (min), median (IQR)	314.50 (213.75–506.25)	314.00 (195.00–360.00)	525.00 (280.00–898.00)	0.113
**Stroke etiology**, ***n*** **(%)**				
Large-artery atherosclerosis	10 (50.0)	5 (38.5)	5 (71.4)	0.350
Cardio-embolism	10 (50.0)	8 (61.5)	2 (28.6)	
Previous rt-PA treatment, *n* (%)	13 (65.0)	9 (69.2)	4 (57.1)	0.651
good collateral status, *n* (%)	20 (100.0)	13 (100.0)	7 (100.0)	/
Successful reperfusion, *n* (%)	19 (95.0)	13 (100.0)	6 (85.7)	0.350
sICH, *n* (%)	1 (5.0)	1 (7.7)	0 (0.0)	1.000
**Location of the occlusive artery**, ***n*** **(%)**				
anterior circulation	18 (90.0)	12 (92.3)	6 (85.7)	1.000
posterior circulation	2 (10.0)	1 (7.7)	1 (14.3)	
mRS on 3 month, median (IQR)	1 (1–4)	1 (0–2)	4 (2–5)	0.061
mRS 0–2 on 3month, *n* (%)	13 (65.0)	10 (76.9)	3 (42.9)	0.174
**Laboratory parameters, median (IQR)**				
CRP, mg/L	11.01 (4.72–11.44)	11.01 (4.39–11.01)	6.74 (4.3–21.92)	0.659
WBC, 10^9^/L	8.10 (6.80–11.58)	7.00 (6.80–9.30)	9.70 (7.30–14.20)	0.104
RBC, 10^12^/L	4.76 (4.05–5.13)	4.86 (4.03–5.30)	4.67 (4.10–4.91)	0.451
PLT, 10^9^/L	221.50 (190.50–273.50)	209.00 (180.50–287.00)	222.00 (201.00–240.00)	1.000
HB, g/L	146.00 (120.25–158.75)	147.00 (127.50–160.50)	143.00 (115.00–155.00)	0.606
Glu, mmol/L	7.98 (6.50–10.80)	7.69 (6.19–9.55)	8.27 (6.82–16.72)	0.405
HBA1c, %	6.75 (5.88–7.38)	6.90 (5.90–7.35)	6.30 (5.80–9.10)	0.968
TG, mmol/L	1.28 (0.98–1.96)	1.28 (0.99–1.83)	1.29 (0.81–1.97)	0.905
TC, mmol/L	4.81 (3.80–5.34)	4.63 (3.70–5.33)	5.19 (4.77–5.36)	0.166
HDL-C, mmol/L	1.02 (0.91–1.13)	1.01 (0.88–1.12)	1.02 (0.90–1.16)	0.751
LDL-C, mmol/L	3.16 (2.60–3.57)	2.98 (2.42–3.64)	3.44 (3.16–3.54)	0.191
CRE, mmol/L	71.00 (53.75–83.00)	69.00 (58.00–78.00)	73.00 (51.00–103.00)	0.721
Hcy, mmol/L	12.47 (9.19–15.26)	10.12 (8.66–13.48)	14.80 (12.50–17.34)	0.096

### Differential expression of mRNA among ENI classes

**Figure 2:**
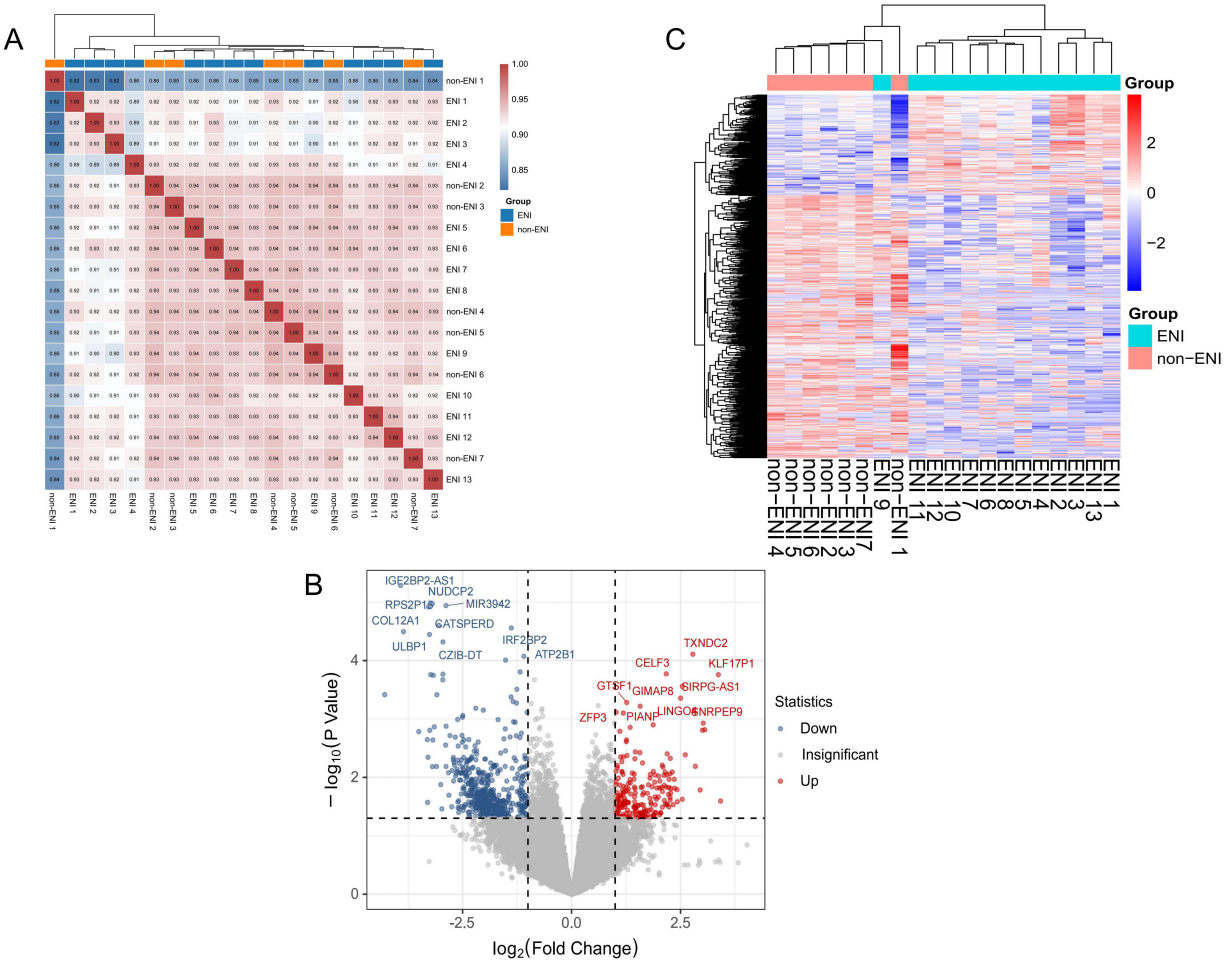
**(A)** Correlation heat map of gene expression levels between each sample. The intensity of the color denotes the magnitude of the correlation coefficient, with blue indicating low correlation and red indicating high correlation. Hierarchical clustering revealed a distinct grouping among the samples, samples from the ENI group exhibited high correlation, whereas samples from the control group were more dispersed in the cluster analysis. **(B)** Volcano plots of genes expression between ENI and non-ENI groups, with red and blue points indicating significantly upregulated and downregulated genes, respectively, and gray points indicating no significant change. (|log2FoldChange| ≥ 1 and P < 0.05). **(C)** Heatmap of significant differentially express genes in between groups by ENI and non-ENI, with rows representing significant differentially expression genes and columns representing samples. Both samples and gene expression levels are hierarchically clustered. Red denotes higher expression levels, whereas blue signifies lower expression levels.

In the 20 samples, 20,501 genes were detected across all samples after adjusted. The Pearson correlation coefficient was employed to quantify the correlation of gene expression levels between samples, and a heat map was constructed for visual analysis. The findings indicated that samples within the ENI group exhibited a higher degree of correlation, whereas those in the non-ENI group were more dispersed. The heatmap (Figure 2A) demonstrated that ENI classes could be grouped based on the expression of these genes.

We identified and visualized 752 significantly differentially expressed genes (DEGs), including 208 upregulated and 544 downregulated genes, using volcano plots, which underscored the distinctions between the ENI and non-ENI groups (Figure 2B). To facilitate a comprehensive analysis of the clustering relationships among samples and the co-expression relationships among genes, we employed hierarchical clustering on the 752 DEGs identified in the prior analysis. This was visualized through a hierarchical clustering heat map (Figure 2C), which elucidates distinct expression patterns of these genes in the ENI group compared to the non-ENI group. These observed patterns may suggest the activation or inhibition of various biological pathways associated with early neurologic improvement.

The ENI group exhibited the most significant enrichment for IGHV1-69D (FC = 10.70, *P* = 0.025). Additionally, this group demonstrated a markedly higher abundance of several genes, including KLF17P1 (FC = 10.33, *P* < 0.001), MEP1B (FC = 8.33, *P* = 0.002), SNRPEP9 (FC = 8.12, *P* = 0.001), and LINC01629 (FC = 8.01, *P* = 0.002). Conversely, in samples associated with the non-ENI group, we observed significantly greater enrichment of C10orf55 (FC = 0.05, *P* < 0.001), IGF2BP2-AS1 (FC = 0.07, *P* < 0.001), COL12A1 (FC = 0.07, *P* < 0.001), KCNK17 (FC = 0.09, *P* = 0.002), and PLAU (FC = 0.10, *P* = 0.002) compared to the ENI group (Supplemental Table 1).

### Bioinformatics analysis of mRNA transcriptomic data

During our GO analysis of the functional roles of DEGs, we found that these genes were differentially enriched in biological processes (BP) such as “regulation of dendritic spine development,” “dendrite development,” “dendritic spine development,” “regulation of leukocyte cell-cell adhesion,” “positive regulation of synaptic transmission, glutamatergic,” “regulation of dendritic spine morphogenesis,” “mechanoreceptor differentiation,” “modulation of chemical synaptic transmission,” “regulation of trans-synaptic signaling,” and “regulation of neuron projection development.”

Correspondingly, molecular function (MF) terms included “lipopeptide binding,” “gated channel activity,” “voltage-gated ion channel activity,” “voltage-gated channel activity,” “cytokine receptor binding,” “NAD+ nucleosidase activity,” “HMG box domain binding,” “coreceptor activity,” “voltage-gated cation channel activity,” and “potassium ion leak channel activity.”

**Figure 3:**
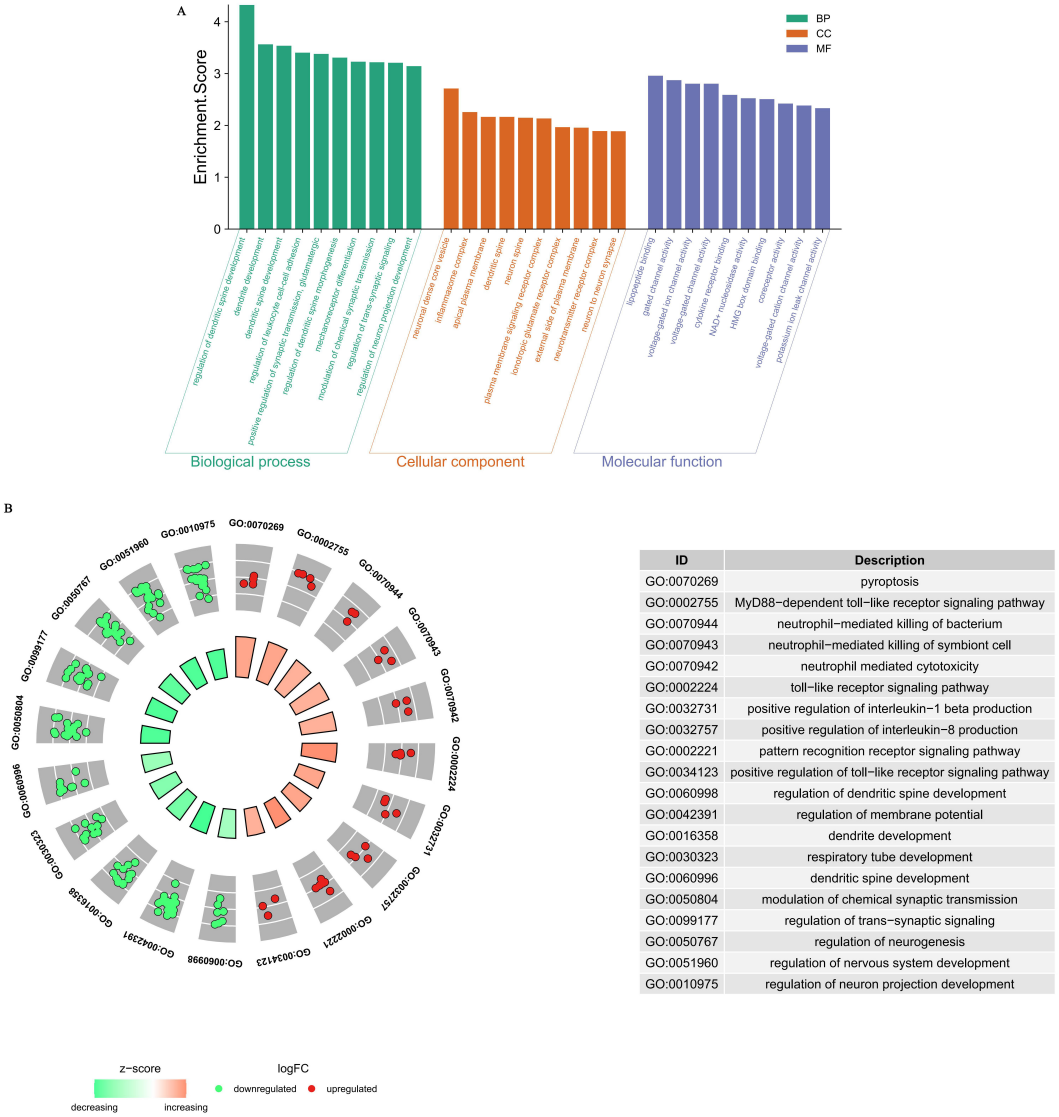
**(A)** Gene Ontology (GO) analysis of significant differentially expression genes. Biological processes (BP) highlight key functional pathways, cellular components (CC) reveal the subcellular localization of the proteins, and molecular functions (MF) depict their biochemical activities. **(B)** BP Enrichment Analysis of Upregulated and Downregulated Genes.

Cellular component (CC) enrichment was mainly observed in neural-specific or immune-related structures, such as “neuronal dense core vesicle,” “inflammasome complex,” “apical plasma membrane,” “dendritic spine,” “neuron spine,” “plasma membrane signaling receptor complex,” “ionotropic glutamate receptor complex,” “external side of plasma membrane,” “neurotransmitter receptor complex,” and “neuron to neuron synapse” (Figure 3A).

To further elucidate the major BP enrichment of upregulated and downregulated genes, we performed GO BP analysis separately. The results showed that the upregulated genes were primarily associated with immune and inflammatory responses, including terms such as “pyroptosis,” “MyD88-dependent toll-like receptor signaling pathway,” “neutrophil-mediated killing of bacterium,” “neutrophil-mediated killing of symbiont cell,” “neutrophil mediated cytotoxicity,” “toll-like receptor signaling pathway,” “positive regulation of interleukin-1 beta production,” “positive regulation of interleukin-8 production,” “pattern recognition receptor signaling pathway,” and “positive regulation of toll-like receptor signaling pathway.” In contrast, the downregulated genes were mainly enriched in BP terms related to neurodevelopment, differentiation, and synaptic function, such as “regulation of dendritic spine development,” “regulation of membrane potential,” “dendrite development,” “respiratory tube development,” “modulation of chemical synaptic transmission,” “regulation of trans-synaptic signaling,” “regulation of neurogenesis,” “regulation of nervous system development,” and “regulation of neuron projection development” (Figure 3B).

**Figure 4:**
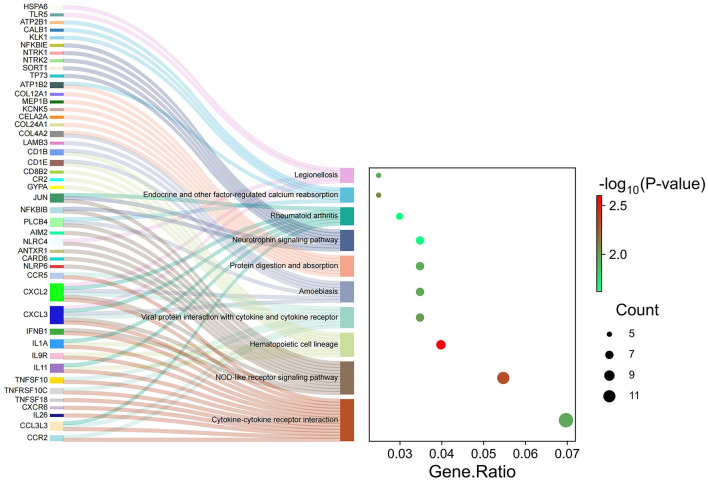
Kyoto Encyclopedia of Genes and Genomes (KEGG) analysis of significant differentially expression genes.

Many signaling pathways were associated with genes of different abundance levels, as revealed by the KEGG signaling pathway enrichment analysis. Significant enrichment was observed in pathways such as “hematopoietic cell lineage” and “NOD-like receptor signaling pathway,” “Endocrine and other factor-regulated calcium reabsorption,” “Viral protein interaction with cytokine and cytokine receptor,” “cytokine-cytokine receptor interaction,” “endocrine and other factor-regulated calcium reabsorption,” “neurotrophin signaling pathway” (Figure 4).

**Figure 5:**
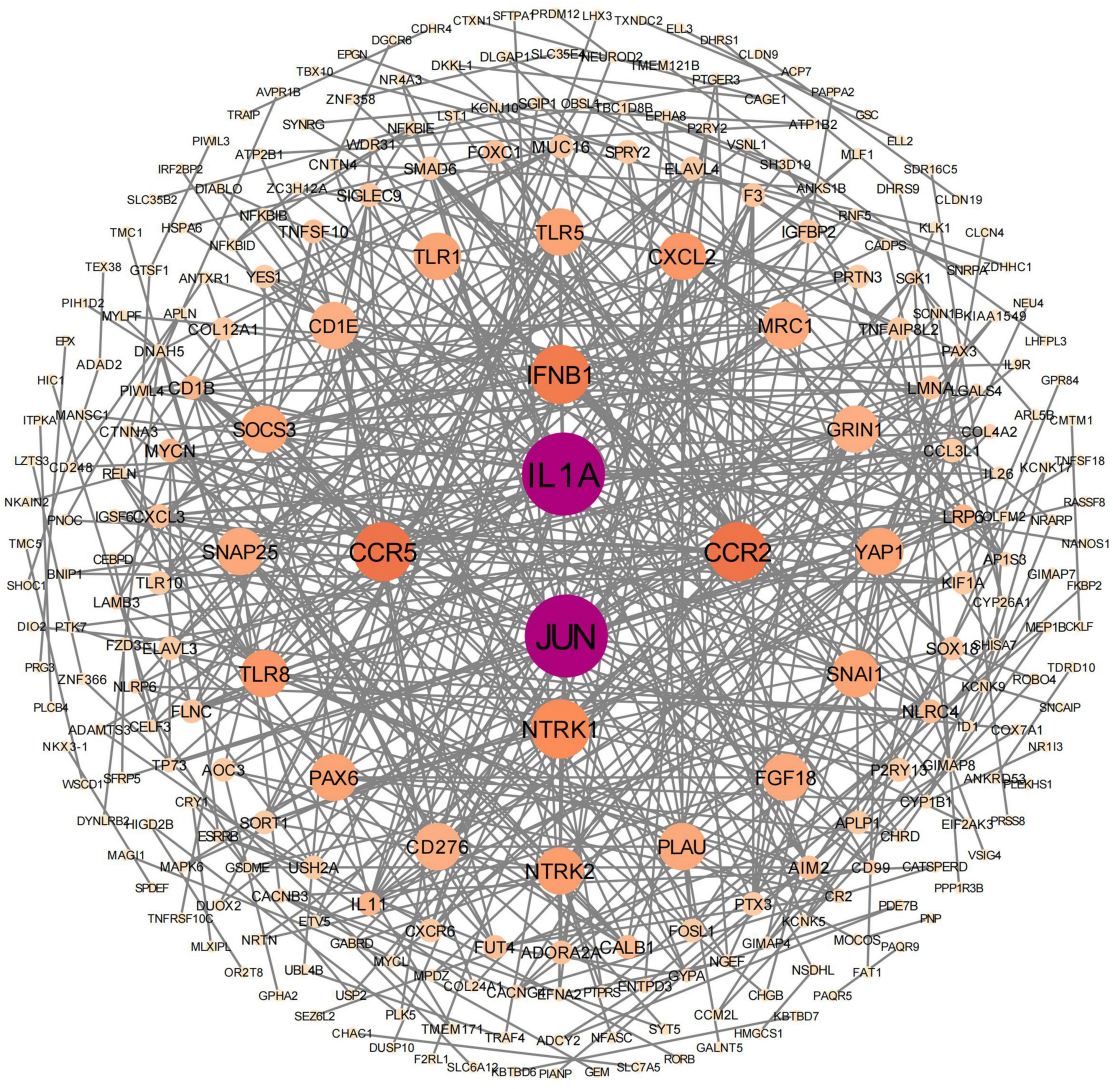
Protein–protein interaction network analysis was conducted on significantly differentially expressed genes. In this network, the nodes represent individual genes that are enriched in the analysis.

PPI analyses were conducted to further examine the cooperative effects of DEGs and to identify potential causal modulation genes (Figure 5). The DEGs were identified as nodes within intricate PPI networks characterized by complex regulatory interactions. The analysis revealed that JUN, IL1A, CCR5, CCR2, IFNB1, NTRK1, TLR8, CXCL2, NTRK2, and GRIN1 are core genes within the top 10 of the network modules. These genes, which play roles in cell growth, differentiation, nervous system development, and immune response, are closely linked with other DEGs.

**Figure 6:**
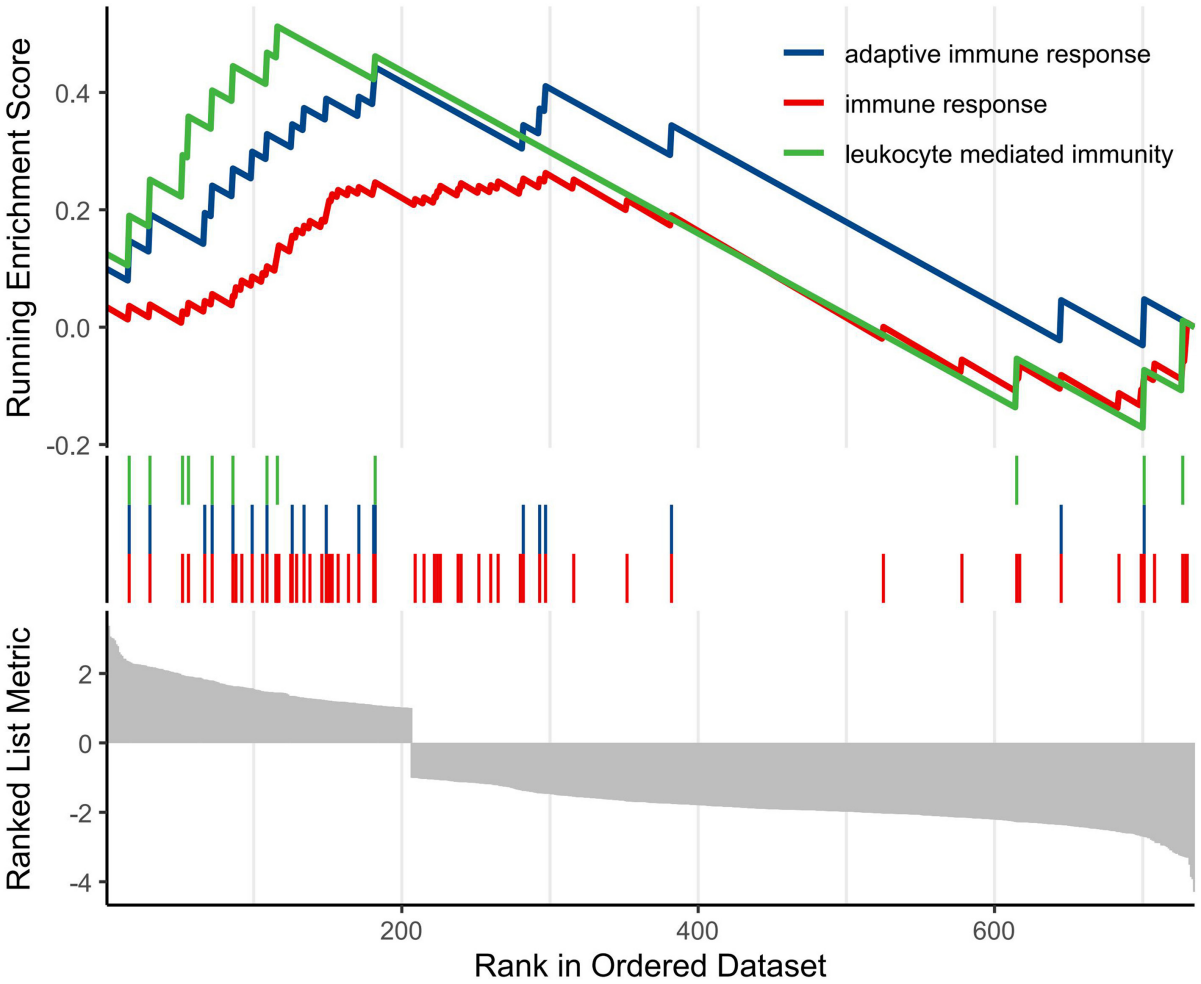
GSEA of biologically relevant pathways based on the GO database.

Finally, we conducted GSEA analysis based on the GO database to screen and visualize biologically meaningful pathways. The analysis demonstrated that the most significantly altered pathways were mainly enriched in processes related to the regulation of adaptive immune responses, general immune responses, and leukocyte-mediated immune functions (Figure 6).

## Discussion

### Overview

By leveraging peripheral blood mRNA transcriptomics, this study systematically compared the molecular profiles of patients with acute ischemic stroke due to large vessel occlusion (AIS-LVO) who exhibited early neurological improvement (ENI) vs. those who did not (non-ENI). Among 20,501 genes detected, 752 were significantly differentially expressed-−208 upregulated and 544 downregulated. Bioinformatics analyses revealed that upregulated genes were primarily enriched in immune and inflammatory response pathways, whereas downregulated genes were mainly involved in neuronal development and synaptic plasticity.

### Upregulation of immune and inflammatory-related genes and pathways: a hypothetical protective inflammatory response

Our results demonstrated a significant upregulation of immune and inflammation-related genes in the peripheral blood of ENI patients during the acute phase of AIS-LVO, implicating several classical inflammatory signaling pathways. These findings may suggest that a moderate inflammatory response could play a protective role in facilitating neurological recovery during the acute stage of stroke, although this hypothesis requires further investigation.

Previous experimental and clinical studies have indicated that the immune system is rapidly activated following stroke, potentially contributing to tissue clearance, removal of necrotic cells, and initiation of repair processes (Iadecola and Anrather, 2011; Chamorro et al., 2012). At the molecular level, upregulation of Toll-like receptors (TLRs), NOD-like receptors (NLRs), chemokines and their receptors (e.g., CCR2, CCR5), inflammasomes, and interleukins (e.g., IL-1β, IL-6, IL-8) may orchestrate orchestrates the recruitment and activation of immune cells (Lambertsen et al., 2012; Gelderblom et al., 2009; Shichita et al., 2012). For example, TLR activation is thought to promote neutrophil and monocyte infiltration to enhance clearance in the lesion area (Lehnardt, 2010), while NLR signaling could induce inflammasome formation and the release of inflammatory mediators such as IL-1β, shaping the local immune microenvironment (Vexler and Yenari, 2009). In this study, upregulation of chemokine receptors CCR5 and CCR2—both linked to inflammatory cell migration—was observed, which might be consistent with findings that CCR5 antagonists can improve neurological outcomes post-stroke (Joy et al., 2020; Jing et al., 2023). Additionally, moderate increases in IL-1 family cytokines may promote reparative immune responses, whereas excessive activation could aggravate secondary injury (Allan et al., 2005; Jayaraj et al., 2019).

Overall, it is hypothesized that “moderate” activation of the inflammatory response may be essential for post-stroke recovery. Immune cells, including neutrophils, macrophages, and T cells, are thought to secrete cytokines and growth factors that could facilitate angiogenesis, promote clearance of necrotic tissue, and stimulate neuronal regeneration (Jin et al., 2010; Xiong et al., 2016). However, excessive or prolonged inflammation may lead to secondary brain injury, blood-brain barrier disruption, and functional impairment (Iadecola and Anrather, 2011). Thus, the intensity and temporal regulation of the immune response during the acute phase might be critical determinants of prognosis (Lambertsen et al., 2012; Chamorro et al., 2012). Our protein-protein interaction (PPI) network analysis identified immune-related genes such as JUN, IL1A, CCR5, CCR2, IFNB1, TLR8, and CXCL2 as central nodes, highlighting their potential roles in modulating inflammation and neural repair. Enrichment analyses (GSEA and KEGG) further confirmed the upregulation of NOD-like receptor, Toll-like receptor, and cytokine-cytokine receptor interaction pathways in the ENI group. These findings appear to align with previous studies and support the hypothesis that a moderate inflammatory response during the acute phase of stroke may be beneficial for neurological recovery, though further validation is needed.

### Downregulation of genes related to neuronal development and synaptic differentiation: protective suppression in the acute phase

In parallel, this study revealed a marked downregulation of genes related to neuronal development and synaptic plasticity in ENI patients during the acute phase. These genes participate in processes such as dendritic spine development, synaptic transmission, and neurogenesis. This transient suppression is hypothesized to represent a protective adaptive mechanism, although the precise implications remain to be fully elucidated.

Previous research suggests that neural repair and functional remodeling mainly occur during the subacute and recovery phases, rather than the acute phase (Carmichael, 2006; Murphy and Corbett, 2009). During acute injury, the brain experiences metabolic stress and energy crisis. Overactivation of neuronal development and synaptic plasticity pathways at this stage could increase energy consumption and excitotoxicity, potentially exacerbating secondary neuronal injury (Dirnagl et al., 1999; Doyle et al., 2008). For example, excessive activation of NMDA receptor-mediated glutamatergic signaling (GRIN1) in early ischemia may lead to intracellular calcium overload and a cascade of apoptotic and necrotic events (Lai et al., 2014; Lipton, 2006). Similarly, while neurotrophic factor receptors such as NTRK1/2 are important for repair, their aberrant activation during acute ischemia might also cause excitotoxic damage (Schäbitz et al., 2007).

Moreover, the suppression of neuronal plasticity during the acute phase may be modulated by inflammatory responses. Inflammatory mediators such as IL-1β and TNF-α are thought to inhibit synapse formation and dendritic differentiation via multiple signaling pathways (Allan et al., 2005; Viviani et al., 2007). Animal studies further suggest that neurogenesis and synaptic remodeling are constrained in regions with active inflammation, with plasticity gradually recovering as inflammation subsides (Jin et al., 2010; Wang et al., 2022). This is considered a potential self-protective mechanism, where temporary inhibition of neuronal activity and synaptic remodeling could reduce energy consumption and excitotoxic risk, creating favorable conditions for subsequent repair (Murphy and Corbett, 2009).

It is noteworthy that the spatiotemporal regulation of neural plasticity post-stroke is likely critical for optimal recovery. Premature or excessive activation of plasticity pathways may be harmful, potentially resulting in abnormal network reconstruction and functional impairments (Carmichael, 2016; Hermann and Chopp, 2012). Therefore, downregulation of neuronal development and synaptic plasticity genes during the acute phase may serve as an “acute protective” mechanism, laying the foundation for neural network reconstruction in later phases, although further studies are warranted to confirm this.

### Integrated perspective and implications

Taken together, these findings suggest that the acute phase of stroke may be characterized by a dynamic balance: upregulation of immune and inflammation-related genes could drive a protective inflammatory response, while downregulation of neuronal development and synaptic plasticity genes might act as a transient protective suppression. This coordinated response is hypothesized to limit secondary injury and create conditions conducive to subsequent neural repair. Understanding these molecular mechanisms may provide new theoretical insights into the regulation of the acute phase of stroke and could inform the optimization of therapeutic intervention timing.

## Conclusion

This study suggests that, during the acute phase of stroke, patients with early neurological improvement (ENI) may exhibit significant upregulation of immune and inflammation-related genes and downregulation of genes related to neuronal development and synaptic plasticity. This pattern might reflect the body's adaptive strategy to acute injury—a dynamic balance between immune activation and plasticity suppression. Moderate immune-inflammatory responses could promote the clearance of necrotic tissue, tissue repair, and neural recovery, while temporary suppression of neuronal development and synaptic plasticity may reduce energy consumption and excitotoxicity, thereby potentially minimizing secondary injury. This dynamic regulation appears to be central to neurological recovery in the acute phase of stroke, although further studies are needed to confirm these observations.

However, several limitations of this study should be acknowledged. First, our study exclusively enrolled patients with good collateral circulation (ASITN/SIR grade ≥2). Previous studies have demonstrated that the status of collateral circulation is not only a critical determinant of prognosis following endovascular therapy in acute ischemic stroke, but also profoundly shapes the distribution of the ischemic penumbra, the immune-inflammatory response, and associated molecular expression patterns (Liebeskind, 2003; Bang et al., 2011). Robust collaterals can slow the progression of the infarct core and improve cerebral perfusion, which in turn may influence gene expression profiles detected in peripheral blood (Christoforidis et al., 2009). Therefore, while restricting enrollment to patients with good collaterals may reduce clinical heterogeneity, it also introduces a selection bias in gene expression characteristics, limiting the generalizability of our findings to patients with poor collateral circulation. Future studies should broaden inclusion criteria and systematically assess gene expression profiles across different collateral statuses to identify biomarkers that are more representative and widely applicable. Second, the relatively small sample size (n=20) inherently restricts the statistical power of our analyses and increases the risk of both type I and type II errors. Moreover, due to the limited number of cases, we were unable to perform comprehensive sensitivity analyses, which may introduce additional bias and affect the robustness of our conclusions. Additionally, whether peripheral mRNA profiles accurately reflect central nervous system events remains uncertain; recent studies suggest that brain-derived exosomal miRNAs may better represent intracranial pathology, especially after blood-brain barrier disruption. We candidly acknowledge these limitations and recognize that they may affect the generalizability and interpretability of our results. Future studies should expand the sample size, include patients with varying collateral circulation statuses, and incorporate approaches such as single-cell sequencing and liquid biopsy to more comprehensively elucidate the molecular characteristics of ENI and enhance the clinical applicability of the research.

## Data Availability

The raw data supporting the conclusions of this article will be made available by the authors, without undue reservation.
