# Supplementary material for: Transcriptomic features of immune inflammation and neural plasticity associated with early neurological improvement in acute ischemic stroke patients with large vessel occlusion
Source: Front Neurosci. 2025 Aug 14;19:1581758. doi: 10.3389/fnins.2025.1581758 (PMC12391103; doi:10.3389/fnins.2025.1581758)
Supplement: Supplementary file 1 [file Table_1.docx]

| **Gene Name** | **logFC** | **AveExpr** | **t** | **P.Value** |
| --- | --- | --- | --- | --- |
| C10orf55 | -4.287344635 | 0.210418975 | -4.268618747 | 0.000383247 |
| IGF2BP2-AS1 | -3.922086412 | -1.459596354 | -6.174361392 | 5.18846E-06 |
| COL12A1 | -3.855896451 | -1.227372833 | -5.349482838 | 3.20449E-05 |
| KCNK17 | -3.506035332 | 2.161619592 | -3.641368562 | 0.001646928 |
| IGHV1-69D | 3.418919352 | 1.207813127 | 2.417304397 | 0.025419753 |
| KLF17P1 | 3.368913712 | -0.642532685 | 4.607680269 | 0.000174566 |
| PLAU | -3.309346992 | 8.224799713 | -3.505466043 | 0.002254648 |
| PRTN3 | -3.303472235 | -0.711707098 | -2.392191407 | 0.026797268 |
| IL1A | -3.294332906 | 0.450189575 | -3.201261013 | 0.004527107 |
| RPS2P15 | -3.279997001 | -0.942438241 | -5.788129775 | 1.20511E-05 |
| ULBP1 | -3.259523862 | -1.040538141 | -5.300104317 | 3.58199E-05 |
| ETV5-AS1 | -3.252305488 | -0.717873029 | -3.663612817 | 0.001564227 |
| CHRD | -3.233353762 | -0.791302967 | -4.607835199 | 0.000174503 |
| NUDCP2 | -3.194306041 | -1.004061783 | -5.844741867 | 1.06388E-05 |
| GSC | -3.192376374 | 0.424648164 | -3.038556892 | 0.006541651 |
| 5_8S_rRNA | -3.18228827 | 0.231178907 | -3.498736765 | 0.002289906 |
| RNU6-375P | -3.170943712 | -0.805719277 | -4.597468912 | 0.000178736 |
| SEMA3F | -3.150081806 | 0.467250938 | -3.722891599 | 0.001363382 |
| RNVU1-15 | -3.100578036 | 1.365444649 | -3.041854168 | 0.006493296 |
| ANXA10 | -3.089819408 | -0.984965948 | -4.266193276 | 0.000385414 |
| MEP1B | 3.05808852 | 0.139635358 | 3.672795194 | 0.001531299 |
| CATSPERD | -3.043370465 | -1.1828108 | -5.461067403 | 2.49379E-05 |
| SNRPEP9 | 3.020792717 | -0.868811332 | 3.785596462 | 0.00117872 |
| LINC01629 | 3.002560146 | -0.034596435 | 3.665462529 | 0.001557538 |
| MLXIPL | -3.002210699 | 1.257133724 | -2.719667366 | 0.013278385 |
| SNCAIP | -2.976002222 | -0.937302307 | -2.269358831 | 0.034588717 |
| LINC00390 | -2.954569871 | -0.777540416 | -4.520692757 | 0.000213491 |
| SOX18 | -2.951820864 | -1.315273993 | -4.615849583 | 0.0001713 |
| CZIB-DT | -2.951552053 | -1.554474701 | -5.170161703 | 4.80717E-05 |
| NPIPA1 | 2.950712994 | 1.654715287 | 2.621750178 | 0.016429569 |
| LINC00511 | -2.889790893 | 0.060747808 | -3.412439533 | 0.002793138 |
| MIR3942 | -2.882776548 | -1.293750073 | -5.810943362 | 1.14602E-05 |
| NEU4 | -2.881013233 | 0.916668643 | -3.041311191 | 0.006501235 |
| RHOV | -2.878702522 | 0.408062304 | -3.733866078 | 0.001329109 |
| TMEM171 | 2.840006921 | 0.960619376 | 3.044272177 | 0.006458055 |
| GEM | -2.82440962 | 1.123947996 | -2.931264911 | 0.008319423 |
| FAT1 | -2.813227033 | -1.053914028 | -2.525474414 | 0.020207734 |
| LRCOL1 | -2.790388626 | -0.947364066 | -3.572969225 | 0.001929279 |
| TXNDC2 | 2.782091627 | 0.06713183 | 4.958306275 | 7.79131E-05 |
| MYCN | -2.748123851 | 0.502900905 | -2.309497714 | 0.031838265 |
| MROCKI | -2.741167406 | 1.967115425 | -2.969636821 | 0.007635811 |
| OR2T8 | -2.715775281 | -0.829822358 | -3.158634746 | 0.004987308 |
| PAPPA2 | -2.712300803 | -0.25901896 | -3.198621959 | 0.004554355 |
| IFNB1 | -2.70782967 | -0.541604006 | -2.785069503 | 0.011503412 |
| LINC02541 | -2.695482005 | 0.749466214 | -3.113046452 | 0.005529805 |
| CXCL2 | -2.686659311 | 6.442885292 | -3.066060669 | 0.006148742 |
| AXDND1 | -2.668136863 | -0.649464382 | -3.547714465 | 0.002045193 |
| OGFOD2 | -2.661554038 | -0.36599848 | -2.873912759 | 0.009452047 |
| LRRC36 | -2.649837812 | 1.144416304 | -2.921750056 | 0.008497841 |
| WDR73 | -2.629704382 | -0.24588203 | -2.99265728 | 0.007252031 |
| MAGI1 | -2.614359178 | -0.694887705 | -2.952945605 | 0.007926246 |
| LAMB3 | -2.614072623 | 7.548227652 | -3.848935378 | 0.001017422 |
| RPL13P4 | -2.61157128 | -0.356501288 | -3.152537757 | 0.005056746 |
| KIF1A | -2.610621565 | 0.702103813 | -2.397546133 | 0.026497851 |
| MDS2 | 2.610112453 | -1.135753503 | 3.24354779 | 0.004111558 |
| RORB | -2.605980184 | 1.366640426 | -2.293659931 | 0.032898511 |
| CPA6 | -2.600941991 | -1.045374205 | -3.394816565 | 0.002908537 |
| CDHR4 | -2.590279816 | 0.682346343 | -2.200388416 | 0.039829606 |
| RNU4-2 | -2.589842984 | -0.09492782 | -2.424674843 | 0.025028155 |
| OSBPL10-AS1 | -2.579075996 | 1.092530881 | -2.278520711 | 0.033942316 |
| CHRFAM7A | -2.569808938 | 0.898642828 | -3.09530116 | 0.005756109 |
| CCL3L3 | -2.569502277 | -0.182226334 | -2.798937939 | 0.011157296 |
| SEZ6L2 | -2.56079808 | -0.854641605 | -2.924021305 | 0.00845492 |
| PCDHGC3 | -2.544857024 | 1.711220316 | -2.80054187 | 0.011117914 |
| SIRPG-AS1 | 2.541583978 | 2.318579567 | 4.408782296 | 0.000276738 |
| FAM157D | 2.540511824 | 4.578164848 | 2.450003172 | 0.023724888 |
| DUT-AS1 | -2.537409946 | 0.315355473 | -2.932210767 | 0.008301886 |
| RPL7AP19 | -2.53388565 | 0.6957889 | -2.314567016 | 0.031505622 |
| MEX3A | -2.533281291 | 0.148044506 | -2.547560671 | 0.019274885 |
| SIGLEC10-AS2 | -2.528310164 | -0.698951579 | -2.922070461 | 0.008491774 |
| DUOX2 | -2.527620104 | 0.156727934 | -3.155591251 | 0.005021853 |
| MTCO2P27 | -2.515112788 | -1.153352328 | -3.576111173 | 0.001915319 |
| ATP5MC2P4 | -2.50887035 | 0.253929009 | -3.02968124 | 0.006673552 |
| AARD | -2.507295816 | -1.472325174 | -3.459901721 | 0.002504249 |
| LINC02770 | -2.504244107 | -1.700451154 | -3.885862755 | 0.000933723 |
| LINGO4 | 2.501879305 | 0.377695102 | 4.208950281 | 0.000440294 |
| CT75 | -2.495543599 | -0.379127295 | -2.64860521 | 0.015501161 |
| ARL14EPL | -2.494187694 | -1.393055497 | -3.915133522 | 0.000872278 |
| U1 | -2.488486332 | -0.818771114 | -2.240399558 | 0.03670701 |
| SNORD3B-1 | -2.486463128 | -0.444345483 | -3.003194059 | 0.007082645 |
| ITGB8-AS1 | -2.482292184 | 0.461052904 | -2.924775003 | 0.008440723 |
| GYPA | -2.474939138 | -1.74457955 | -3.197871399 | 0.004562134 |
| UHRF2P1 | -2.470447203 | -0.159073258 | -3.503416379 | 0.00226533 |
| LINC03068 | -2.46821752 | -0.457234754 | -2.678531081 | 0.01452518 |
| DPY19L2 | -2.467494581 | 2.290782845 | -2.301984878 | 0.032337213 |
| PLET1 | -2.464909058 | 0.468202476 | -2.637574328 | 0.015876338 |
| LRRC37A4P | -2.462080496 | -1.224756772 | -2.322981572 | 0.030960544 |
| WDR31 | -2.450400859 | 0.382658378 | -2.93995392 | 0.008159645 |
| RASSF8 | -2.437885033 | 1.347696981 | -2.555917749 | 0.018932589 |
| ZNF704 | -2.43755595 | 0.943830049 | -2.351669325 | 0.029167045 |
| ICAM4-AS1 | -2.433500297 | -0.989647086 | -2.925818736 | 0.008421101 |
| PIWIL3 | -2.431395976 | -1.504429649 | -2.870823123 | 0.00951709 |
| PLCB4 | -2.428111186 | 0.533852978 | -3.379915449 | 0.003009753 |
| RPS20P22 | -2.427543311 | -1.082243322 | -2.778997167 | 0.011658167 |
| MTX1P1 | 2.427091742 | 3.055952404 | 2.405038056 | 0.026084148 |
| H3C6 | 2.420115617 | 3.431839737 | 2.628510392 | 0.01619103 |
| NEUROD2 | -2.414607246 | -0.162739456 | -3.078892144 | 0.005973349 |
| PMS2P2 | -2.413113609 | -0.490610542 | -2.873729673 | 0.00945589 |
| PAX6 | -2.409691233 | -0.67043095 | -2.554420872 | 0.018993476 |
| ELL3 | -2.406056983 | -0.728628725 | -3.12117055 | 0.005429103 |
| CAGE1 | -2.401232243 | 0.17083138 | -2.862796903 | 0.009688071 |
| UNGP3 | -2.392825765 | -1.448691365 | -3.153863541 | 0.005041567 |
| ANTXR1 | -2.38747554 | -0.66810647 | -2.512763277 | 0.020763663 |
| GPHA2 | -2.382587808 | -0.520042985 | -2.987777125 | 0.007331802 |
| ZFX-AS1 | -2.37333591 | -0.280384187 | -2.728041549 | 0.013037357 |
| CTXN1 | -2.372564707 | -1.359994662 | -2.205200367 | 0.039441625 |
| CAMK2N2 | -2.369281441 | -0.260684523 | -3.003555442 | 0.007076904 |
| SFRP5 | 2.36727084 | 1.179244695 | 2.651505615 | 0.015403917 |
| ANKRD65 | -2.365041397 | -1.480238807 | -2.736151572 | 0.012807904 |
| TRBV20-1 | -2.361554057 | 1.636391602 | -2.401003547 | 0.026306181 |
| LINC01122 | -2.361508026 | -1.111098944 | -3.144728745 | 0.005147056 |
| GDPD2 | -2.356350203 | -1.23325871 | -3.504727664 | 0.00225849 |
| BRD7P3 | 2.355850459 | 0.14433103 | 2.813559868 | 0.010803161 |
| CLEC4OP | -2.355042687 | -0.808234395 | -2.127114282 | 0.046183734 |
| EPGN | -2.350180075 | -0.250039083 | -2.208739291 | 0.039158494 |
| MIR3140 | -2.347149284 | 0.821397766 | -2.522524422 | 0.020335494 |
| HTR1DP1 | -2.345088441 | -0.558604409 | -3.146238009 | 0.00512948 |
| CCM2L | -2.344117061 | 0.581467119 | -2.341290001 | 0.029804535 |
| PSPC1P1 | -2.342619899 | -0.440945094 | -2.930402987 | 0.008335436 |
| MYCNOS | -2.336842755 | -1.394655707 | -2.135017687 | 0.045456598 |
| ATRNL1 | -2.332913231 | -0.531478859 | -2.616151589 | 0.016629631 |
| CD1E | 2.332247948 | 2.425261166 | 2.349570444 | 0.029294929 |
| LINC00487 | -2.325656201 | 0.111661391 | -2.747740365 | 0.012486692 |
| ADAM7-AS1 | -2.32427087 | 0.021733978 | -2.519129451 | 0.020483464 |
| ANKRD18CP | -2.313337798 | -0.423210774 | -2.779859195 | 0.011636078 |
| ACP7 | -2.312738931 | -1.778059294 | -3.170409896 | 0.004855817 |
| FGF18 | -2.311972541 | 0.025513607 | -2.813692224 | 0.010800005 |
| HAUS1P1 | 2.311938867 | 1.77164406 | 2.943766309 | 0.008090477 |
| ATP1B2 | -2.306778111 | 0.523229999 | -2.602421274 | 0.017130062 |
| FAM9C | -2.301208716 | -0.692483181 | -2.518597935 | 0.020506722 |
| PERM1 | -2.29933228 | -0.601870746 | -2.708115198 | 0.013617848 |
| SEPTIN7P12 | -2.299063925 | -0.308787759 | -2.773411558 | 0.01180227 |
| LINC02739 | -2.296596717 | -0.609332421 | -3.18286039 | 0.004720455 |
| FLNC | -2.295676187 | -0.312284531 | -2.380642751 | 0.02745376 |
| F3 | -2.29196646 | 3.612009471 | -2.317798886 | 0.031295228 |
| PAQR5 | -2.291855354 | 0.01622453 | -2.796179596 | 0.011225334 |
| VDAC2P3 | -2.291124692 | 0.161368505 | -2.61164768 | 0.016792243 |
| ESRRB | -2.291111993 | 0.623859302 | -2.643156388 | 0.015685422 |
| DKKL1 | -2.287956043 | -1.06985072 | -3.212469184 | 0.004413139 |
| CHGB | -2.286405549 | -0.992996965 | -2.402300639 | 0.026234606 |
| PRG3 | -2.281961566 | -1.520930455 | -2.664732301 | 0.014967736 |
| RAB6C-AS1 | -2.281224853 | -1.151908463 | -3.010634929 | 0.006965336 |
| MTCYBP35 | 2.28035743 | -0.793077713 | 2.649175529 | 0.015481994 |
| NR4A3 | -2.280155071 | 7.614891029 | -2.21474065 | 0.038682601 |
| ARHGEF16 | -2.275381399 | -0.183915624 | -2.813594836 | 0.010802327 |
| MIR4482 | 2.273834974 | 0.331549582 | 2.567233401 | 0.018478222 |
| MTCO1P31 | 2.266130154 | -1.359341998 | 3.182324677 | 0.004726203 |
| TGM3 | 2.261951867 | -0.93438812 | 2.436645681 | 0.024404092 |
| TMED11P | 2.260807292 | -0.537855349 | 2.488217044 | 0.021878032 |
| RPL12P18 | -2.258956827 | -1.16610764 | -3.028342927 | 0.006693663 |
| ROBO4 | 2.258954584 | -0.90727816 | 2.91203816 | 0.008683747 |
| U3 | -2.257085332 | -0.239206505 | -2.360833852 | 0.028614689 |
| HEPHL1 | -2.254423661 | 0.392058277 | -2.712975146 | 0.013474044 |
| ZRANB2-DT | -2.24324014 | -0.623959355 | -2.499213037 | 0.021372071 |
| CELA2A | -2.241874527 | -0.554112922 | -2.542896615 | 0.019468442 |
| LINC01348 | -2.238618823 | 1.397657677 | -2.570320973 | 0.018356039 |
| WASF4P | 2.237456743 | -0.939996895 | 2.812375204 | 0.010831448 |
| LINC01580 | 2.234697863 | -1.379772987 | 2.307962126 | 0.031939666 |
| RPSAP22 | 2.233809705 | -0.44013277 | 2.664958927 | 0.014960365 |
| EPHA8 | -2.233490812 | -1.414052511 | -3.309173361 | 0.003539353 |
| COX6CP1 | -2.232862701 | 0.211309305 | -2.66396371 | 0.014992758 |
| ITPKA | -2.224894649 | 0.446831522 | -2.432923463 | 0.024596567 |
| IL26 | 2.222550734 | -0.313155994 | 2.539189939 | 0.019623569 |
| LINC02256 | -2.221521943 | -1.212251963 | -2.642034412 | 0.01572362 |
| MUC16 | -2.215435212 | -1.583851927 | -3.399313425 | 0.002878653 |
| APCDD1L-DT | -2.212032818 | -0.640271911 | -2.729200396 | 0.013004332 |
| PPP1R27 | -2.21153888 | -0.607646557 | -2.290083721 | 0.033142378 |
| TXNP6 | -2.211457773 | -1.217481973 | -2.507662502 | 0.020990757 |
| CASKIN1 | -2.208803422 | -0.924195532 | -2.36575802 | 0.028321924 |
| SCGB1C1 | -2.207260825 | 0.370554637 | -2.417732299 | 0.025396863 |
| SYNM-AS2 | -2.201363833 | -0.615494024 | -2.135232649 | 0.045436966 |
| MANSC4 | -2.198992635 | -1.128338702 | -2.81935957 | 0.010665694 |
| HMGN2P48 | 2.198428536 | 0.333411628 | 2.354641967 | 0.028986807 |
| RNFT1-DT | -2.197603565 | -1.834129146 | -4.038304868 | 0.000654929 |
| TMC5 | 2.194071193 | 2.159869159 | 3.260302405 | 0.003957416 |
| LINC00163 | -2.193340263 | -1.550725892 | -3.159197984 | 0.00498094 |
| ZNF358 | -2.192511298 | 0.113920122 | -2.284130839 | 0.033552009 |
| AMZ2P2 | -2.190921542 | 0.149168767 | -2.236096367 | 0.037031743 |
| STAG1-DT | -2.188599748 | -0.895977852 | -2.699013196 | 0.013891105 |
| CD1B | 2.187052509 | 1.381326362 | 2.897992006 | 0.008959551 |
| GRIN1 | -2.185963488 | 0.060373183 | -2.284625769 | 0.033517775 |
| ADAD2 | 2.183421623 | -1.413102543 | 2.348928915 | 0.029334121 |
| NSDHL | -2.179153381 | 0.872124085 | -2.551462377 | 0.019114358 |
| USP12-AS1 | -2.17816103 | -1.447809618 | -3.257890455 | 0.003979253 |
| SNORA2C | -2.177734542 | -0.0711058 | -3.315791695 | 0.003486167 |
| DIRC3 | -2.17729993 | -0.107143472 | -2.450134758 | 0.023718286 |
| KIAA1549 | -2.17482701 | 1.374755155 | -2.342724278 | 0.029715681 |
| CACNG4 | 2.174771597 | -0.477027495 | 2.674268684 | 0.014660542 |
| MIR6746 | -2.172903058 | -1.261406556 | -2.763571413 | 0.01206028 |
| CELF3 | 2.172651304 | 1.457956942 | 4.622043692 | 0.000168865 |
| CRYZL2P | -2.16294392 | 1.171838996 | -2.325761711 | 0.030782375 |
| SYT5 | -2.156934026 | -0.915375028 | -2.373634599 | 0.027859393 |
| FOXC1 | -2.155417557 | 4.931025914 | -2.573709555 | 0.01822282 |
| MIR4742 | -2.155105586 | -0.136911044 | -2.550248238 | 0.019164176 |
| RGS11 | -2.153776188 | -0.814352492 | -2.502918885 | 0.021204038 |
| NBPF4 | 2.153770282 | -1.432375915 | 2.36350994 | 0.028455236 |
| PLEKHS1 | -2.152327504 | -0.538531456 | -2.36577134 | 0.028321135 |
| C11orf96 | -2.147608475 | 0.806043855 | -2.570234544 | 0.018359449 |
| SMAD6 | -2.146405835 | 3.381024321 | -2.550679139 | 0.019146481 |
| PNRC1-DT | -2.145371751 | -0.295305036 | -2.357279563 | 0.028827749 |
| ELAVL3 | -2.145091269 | -0.139710152 | -2.508278157 | 0.020963224 |
| COX7CP2 | -2.144347509 | 1.626562645 | -2.985969886 | 0.007361557 |
| SNORA74C-1 | 2.144224566 | -1.032030425 | 2.94197666 | 0.008122876 |
| SLC25A47P1 | -2.143278634 | 0.256623945 | -2.593056462 | 0.017479502 |
| UBE2V1P1 | -2.142941761 | 2.099204037 | -3.464102165 | 0.002480145 |
| EFNA2 | -2.141402447 | -1.025215448 | -2.483158417 | 0.022114543 |
| SHD | -2.134908809 | 2.678333551 | -2.476628802 | 0.022423368 |
| ENO1-AS1 | -2.133412735 | -0.632722275 | -2.933279914 | 0.008282104 |
| CSPG4BP | -2.129842982 | 0.117650623 | -2.497649546 | 0.021443338 |
| MIR3150BHG | 2.129145501 | 0.138444054 | 2.428351073 | 0.024834941 |
| ABCA12 | -2.128136665 | -0.863810261 | -2.928434492 | 0.008372118 |
| LGALS4 | 2.125187472 | -1.450954741 | 2.705049927 | 0.013709297 |
| NGEF | 2.124604178 | -0.12895334 | 2.885146042 | 0.009219141 |
| SHC1P2 | -2.123355723 | 0.030668471 | -2.29514241 | 0.032797905 |
| CADPS | -2.121691319 | -1.695340714 | -2.923746957 | 0.008460094 |
| AQP8 | -2.12147203 | -0.548163119 | -2.331260396 | 0.030432772 |
| FAM83A | -2.117168831 | -1.049301223 | -2.270170824 | 0.034530974 |
| BNIP3P27 | -2.116796266 | 0.461301735 | -2.628206426 | 0.016201685 |
| GTF3C2-AS1 | -2.110666818 | -0.663111204 | -2.51583121 | 0.020628186 |
| CD81-AS1 | -2.10812785 | 0.80900758 | -2.449647547 | 0.02374274 |
| AP1S3 | -2.105540847 | 2.025126517 | -2.237241171 | 0.036945096 |
| TEX38 | -2.105252926 | -1.017253157 | -2.838608332 | 0.010221348 |
| GOT2P2 | -2.103771964 | -0.054625277 | -2.335020602 | 0.030195818 |
| UNC93B3 | -2.099002792 | -1.356729339 | -2.376227537 | 0.027708669 |
| LDHAL6A | 2.09868268 | 1.53729896 | 3.029541706 | 0.006675646 |
| WSCD1 | -2.092046651 | -0.836330779 | -2.158216084 | 0.043381882 |
| LINC03008 | -2.091935922 | -0.858287663 | -2.430403153 | 0.024727694 |
| ZSCAN31 | -2.090281276 | 0.87696343 | -2.377866798 | 0.027613772 |
| AVPR1B | -2.090171882 | -1.017663429 | -2.153854504 | 0.043765259 |
| APLP1 | -2.089277059 | 1.085885712 | -2.395206861 | 0.02662827 |
| RPL17P38 | -2.087633962 | -1.549568735 | -2.550941372 | 0.01913572 |
| RNU6-807P | 2.084257037 | 0.698693917 | 2.59271526 | 0.01749236 |
| CCDC192 | 2.082816307 | 0.329138522 | 2.302044999 | 0.032333192 |
| HSPD1P1 | -2.080770524 | 1.555912054 | -2.50539772 | 0.021092333 |
| LINC02289 | 2.079773825 | 5.340755689 | 3.063678337 | 0.00618185 |
| HNRNPA1P16 | -2.069653223 | 1.133607689 | -2.349711949 | 0.029286291 |
| GABRD | -2.069591638 | 0.58597653 | -2.167320309 | 0.042591458 |
| CTNNA3 | -2.067091218 | -1.342843827 | -2.754372599 | 0.012306331 |
| XACT | -2.066332771 | -1.825192046 | -2.943292814 | 0.008099037 |
| NRBF2P2 | 2.065192521 | -0.61275227 | 2.667684736 | 0.014871983 |
| C20orf203 | -2.063216927 | -1.826282591 | -2.775498215 | 0.011748239 |
| ENTPD3 | -2.06220322 | -0.277843947 | -2.593692615 | 0.017455553 |
| EIF5-DT | -2.058652891 | 1.711203711 | -2.309022857 | 0.03186959 |
| NKAIN2 | -2.058049325 | -0.327136527 | -2.568262578 | 0.01843741 |
| GPC1-AS1 | -2.056976705 | -1.338744675 | -2.364451421 | 0.028399335 |
| TUSC3 | -2.052950455 | -1.560966972 | -2.487425371 | 0.021914889 |
| FAM83E | -2.051605606 | -0.118454361 | -2.265640902 | 0.034854243 |
| ADAMTS3 | -2.050912173 | -1.401085863 | -2.321905983 | 0.031029729 |
| CSF2RBP1 | 2.047810354 | 3.88482767 | 2.194438282 | 0.040314175 |
| CYP26A1 | -2.047310257 | 0.048292728 | -2.403665061 | 0.026159512 |
| ZNF165 | -2.046944388 | 3.130741551 | -2.914722054 | 0.008631984 |
| DQX1 | -2.046788249 | 1.439790106 | -2.418778277 | 0.025340991 |
| ANKRD53 | -2.044954189 | 2.594616693 | -2.462996566 | 0.023081167 |
| SNHG25 | -2.043812841 | -0.991728966 | -2.605246795 | 0.017025932 |
| MIR3662 | -2.043622763 | 0.332586376 | -2.196780436 | 0.040122795 |
| MIR548P | 2.042632625 | -0.502951617 | 2.193320543 | 0.0404058 |
| SNRPGP18 | -2.042378038 | 0.45719207 | -2.335341034 | 0.030175705 |
| CLDN19 | -2.039929402 | -0.987886435 | -2.538807389 | 0.019639645 |
| MIR193BHG | -2.039000058 | -1.522113419 | -2.802083195 | 0.011080195 |
| C9orf163 | -2.038220883 | -0.213082797 | -2.351832789 | 0.029157107 |
| IGSF22-AS1 | -2.037497152 | -0.528561665 | -2.489534861 | 0.021816808 |
| RCOR2 | -2.037023211 | -0.432799148 | -2.114918347 | 0.047326499 |
| IL11 | -2.035605084 | -0.510386665 | -2.213552142 | 0.038776424 |
| APLN | -2.030853828 | -1.257340297 | -2.528216026 | 0.020089674 |
| THSD8 | -2.030320193 | 0.688866726 | -2.130685038 | 0.045853918 |
| PARLP1 | -2.028553159 | -0.030927225 | -2.497229149 | 0.021462538 |
| H2AC21 | -2.027249939 | 0.187219274 | -2.296923425 | 0.032677413 |
| SRSF6P2 | -2.027081842 | -0.272198577 | -2.50837969 | 0.020958687 |
| LINC01119 | 2.02558249 | 0.111087323 | 2.783252442 | 0.011549514 |
| SOCS5P2 | -2.024751919 | -2.123663427 | -4.005428092 | 0.000707004 |
| IFITM3P8 | 2.023094054 | -0.562169239 | 2.15402998 | 0.043749775 |
| RPL36P11 | 2.020796117 | -0.672743055 | 2.657612035 | 0.015201068 |
| RPL36AP19 | -2.020107275 | -1.198798885 | -2.638889395 | 0.015831163 |
| ACOT4 | -2.019771468 | -0.952575379 | -2.549902273 | 0.019178394 |
| SNORA54 | -2.014867174 | -0.748976509 | -2.492070179 | 0.021699473 |
| SAMD11P1 | -2.014567765 | -0.976195173 | -2.611863268 | 0.016784426 |
| EFCAB10 | -2.010216902 | 0.379101708 | -2.172296724 | 0.04216497 |
| PKD1P1 | -2.009858187 | -1.258586625 | -2.744933267 | 0.012563786 |
| DPY19L3-DT | -2.007756705 | -1.295467968 | -2.93199201 | 0.008305939 |
| TSACC | -2.006926715 | -0.597362223 | -2.567518259 | 0.018466918 |
| MTND5P1 | -2.005120624 | 0.293345355 | -2.163730515 | 0.042901543 |
| LHFPL3 | -2.003281171 | 0.097046925 | -2.607417385 | 0.016946344 |
| PTGER3 | -2.000621308 | 4.932006705 | -2.854735826 | 0.009862756 |
| LINC02591 | 1.999574565 | 1.560180278 | 2.872603398 | 0.00947956 |
| AFF4-DT | -1.997899832 | -0.898822415 | -2.320084349 | 0.031147229 |
| RPL12P44 | -1.997714623 | 0.28437457 | -2.416545016 | 0.025460423 |
| RNY3P12 | -1.991538511 | -1.913769769 | -3.169216115 | 0.004868993 |
| OR52I1 | 1.990804121 | -0.574381186 | 2.495739551 | 0.0215307 |
| SKOR1-AS1 | -1.988741585 | -1.115186072 | -2.29509787 | 0.032800923 |
| EGOT | -1.988528431 | -0.930370026 | -2.462081532 | 0.02312596 |
| CNTN4 | -1.986729572 | -1.066185179 | -2.321307199 | 0.031068307 |
| NRTN | -1.983464573 | 0.111623999 | -2.271326018 | 0.034448979 |
| PIH1D2 | -1.981087056 | -0.619682891 | -2.126335621 | 0.046255941 |
| MTND4LP7 | -1.979886564 | -1.005847384 | -2.562945927 | 0.018649159 |
| MFSD1P1 | -1.979529071 | 0.626945739 | -2.198310284 | 0.039998237 |
| RPS27AP8 | -1.978999003 | -0.867982368 | -2.22124024 | 0.03817317 |
| MRPS24 | -1.9780875 | -1.255989135 | -2.327900579 | 0.030645948 |
| HMGB3P14 | -1.977393475 | 1.216173279 | -2.172233889 | 0.042170331 |
| TMC1 | -1.975362835 | -1.667877259 | -3.17112907 | 0.004847896 |
| NANOS1 | -1.974521265 | -1.303502321 | -2.414518296 | 0.025569262 |
| SNORD12B | -1.972960297 | -0.106087711 | -2.510433543 | 0.020867099 |
| RNU6-101P | -1.971081537 | 0.530124356 | -2.626945351 | 0.01624596 |
| PLPPR3 | -1.969854736 | 4.078035182 | -2.325792885 | 0.030780383 |
| YWHAZP10 | -1.969128106 | -0.127960407 | -2.289859024 | 0.033157756 |
| HMGN1P38 | -1.96780146 | 1.352276688 | -2.415459974 | 0.025518638 |
| LNMICC | -1.96715635 | 0.150790751 | -2.624751635 | 0.016323253 |
| SUPT20HL2 | -1.965296758 | -1.464341655 | -2.182952344 | 0.041264819 |
| FAM204BP | -1.962926559 | -1.290564058 | -2.505954039 | 0.02106734 |
| QRSL1P3 | -1.960054685 | -0.66533933 | -2.322806899 | 0.03097177 |
| MIR1537 | -1.95927964 | -1.315638196 | -2.461204192 | 0.023168985 |
| ACTG1P22 | -1.957552855 | -0.047795853 | -2.087669156 | 0.049972736 |
| LINC01729 | 1.956200147 | -0.721557795 | 2.657493354 | 0.015204986 |
| NTRK2 | -1.953388449 | -0.015358394 | -2.43760582 | 0.024354672 |
| DNM1P33 | -1.951043445 | 0.284392824 | -2.099802109 | 0.048778392 |
| LHX3 | -1.949707305 | -0.127158404 | -2.52299425 | 0.020315096 |
| FGD1 | -1.949171418 | 0.021736403 | -2.111240595 | 0.047676096 |
| SCNN1B | 1.948942222 | -1.153945211 | 2.45313625 | 0.023568153 |
| ELAVL4 | -1.947520807 | -0.141534622 | -2.220482844 | 0.038232216 |
| RPL21P83 | -1.943931436 | -1.221319856 | -2.283400923 | 0.033602556 |
| SPATA13-AS1 | -1.943118887 | 0.028421831 | -2.355950432 | 0.0289078 |
| MOCOS | -1.939253681 | 0.114465032 | -2.531295295 | 0.019957846 |
| CSRP1-AS1 | -1.939126867 | -0.075985343 | -2.143272363 | 0.044708246 |
| GTF2I-AS1 | -1.939036487 | 0.455770188 | -2.699267114 | 0.013883412 |
| RANBP17 | -1.938509822 | 0.553830476 | -2.179404185 | 0.041562591 |
| H2BP1 | 1.93734632 | -0.396337197 | 2.200527233 | 0.039818364 |
| RPS12P4 | -1.936478233 | -0.806911239 | -2.485274898 | 0.022015299 |
| SLC25A47 | -1.936472827 | 0.573460404 | -2.231607682 | 0.037373277 |
| LINC01505 | -1.935750659 | 0.474777759 | -2.149741896 | 0.04412957 |
| UBE2Q2P13 | -1.932559536 | -0.321298058 | -2.798130332 | 0.011177176 |
| TFAP2D | -1.932371818 | -1.87207838 | -2.864589477 | 0.00964963 |
| LIMS1-AS1 | -1.931366749 | 1.035552085 | -2.162888816 | 0.042974546 |
| CCT5P1 | -1.93127941 | -0.473573286 | -2.098161216 | 0.048938395 |
| CCDC28A-AS1 | -1.931242359 | -1.057925705 | -2.139118701 | 0.045083399 |
| RNU4-5P | -1.929579317 | -0.411016766 | -2.217931641 | 0.038431722 |
| PRELID3A | -1.928602215 | 2.169186515 | -2.365439146 | 0.028340798 |
| HNRNPA1P45 | -1.927760743 | -1.57518407 | -2.710930118 | 0.013534379 |
| TDRD10 | -1.923453834 | -0.328820183 | -2.407344908 | 0.025957979 |
| IGFL2 | 1.921086711 | -1.583620236 | 2.345405478 | 0.029550238 |
| SNX18P12 | -1.920934532 | -1.850131707 | -2.806385399 | 0.010975558 |
| RRH | -1.920640728 | 0.289685724 | -2.095662681 | 0.049182939 |
| KLF3P1 | -1.920391841 | -1.304426093 | -2.405410573 | 0.026063735 |
| NUDT9P1 | -1.92032273 | 0.725393244 | -2.306709371 | 0.032022612 |
| SHISA7 | -1.919709601 | -0.228996076 | -2.247736427 | 0.036159362 |
| SNAP25 | -1.918441029 | -0.69189347 | -2.27249714 | 0.034366035 |
| CATIP-AS2 | -1.918363287 | -1.008109477 | -2.156066453 | 0.043570448 |
| RNU6-469P | 1.915714718 | 0.534223386 | 2.494465977 | 0.021589139 |
| ETV5 | -1.914969652 | 3.828910165 | -2.888333026 | 0.009154073 |
| USP2 | -1.914564218 | 0.462317313 | -2.118099465 | 0.047025984 |
| F2RL1 | 1.914085271 | 7.577719034 | 2.892404231 | 0.009071593 |
| SGIP1 | -1.911357478 | 0.181306294 | -2.094598381 | 0.049287442 |
| PCDHGC5 | -1.910802375 | 0.689410131 | -2.429678833 | 0.024765499 |
| RTCA-AS1 | -1.910395178 | 0.566003107 | -2.14084047 | 0.044927546 |
| LINC02312 | 1.910025918 | -0.657419592 | 2.196568314 | 0.040140093 |
| HMGB1P23 | -1.90917753 | -1.840707866 | -2.753216579 | 0.012337589 |
| PKIA-AS1 | -1.907671124 | 0.321739774 | -2.275550096 | 0.034150675 |
| TBC1D8B | 1.906861948 | 3.040932813 | 2.76390822 | 0.01205136 |
| TM4SF19-AS1 | -1.90413145 | -0.44362292 | -2.260456025 | 0.035227664 |
| KCNH4 | -1.903768373 | 2.9046418 | -2.871804974 | 0.009496374 |
| HAUS6P1 | -1.900383548 | -1.128831194 | -2.359845993 | 0.028673759 |
| LCMT1-AS1 | -1.900146573 | -0.800517826 | -2.394041351 | 0.026693472 |
| CYTL1 | -1.898398934 | 1.928297119 | -2.148526355 | 0.044237773 |
| PPIAL4C | 1.898367999 | -0.137841033 | 2.131649035 | 0.045765244 |
| ADORA2BP1 | -1.896706719 | -1.077949933 | -2.546486387 | 0.019319307 |
| NRARP | -1.891519868 | 2.915828473 | -2.406854379 | 0.025984759 |
| H4C1 | -1.891367381 | 1.088626128 | -2.378355876 | 0.027585518 |
| PPP1R12A-AS1 | -1.891031818 | -0.425884468 | -2.179710512 | 0.041536806 |
| CALCRL-AS1 | -1.888074604 | -1.887582405 | -2.12640716 | 0.046249303 |
| MPHOSPH10P1 | 1.885291948 | 2.09926652 | 2.156418537 | 0.043539512 |
| PRR35 | -1.88156646 | -1.350173054 | -2.420247738 | 0.025262691 |
| RNA5SP78 | -1.880772454 | -0.084805694 | -2.463757185 | 0.023043995 |
| LINC01002 | 1.880255937 | 4.193864521 | 2.131211093 | 0.045805509 |
| OR52I2 | 1.879481128 | -0.635251006 | 2.140035222 | 0.045000375 |
| DYNLRB2 | -1.879409069 | -0.764927002 | -2.257342279 | 0.035453682 |
| TENM2 | -1.879255048 | -1.518715394 | -2.406989506 | 0.02597738 |
| CRACDL | 1.876370596 | 5.856074178 | 2.95402542 | 0.007907139 |
| YPEL3-DT | 1.872808575 | 3.232212496 | 3.757704625 | 0.001257577 |
| NPEPPSP1 | -1.868810641 | -0.151439081 | -2.107355707 | 0.048047919 |
| RNU6-583P | -1.868718159 | -1.82729153 | -2.59454495 | 0.017423514 |
| SPDEF | -1.866788791 | 1.824484416 | -2.122115805 | 0.046649036 |
| ADORA2A | -1.865521052 | 6.8070312 | -2.541802798 | 0.019514099 |
| PLCB2-AS1 | -1.863679516 | -0.767786269 | -2.280487768 | 0.033804991 |
| BAALC-AS1 | -1.85996336 | 0.04245904 | -2.576764614 | 0.018103495 |
| KRT18P15 | -1.857159604 | 0.028555714 | -2.214462105 | 0.038704571 |
| EPOP | -1.855978376 | 1.930311283 | -3.103227412 | 0.005653937 |
| IGFBP2 | -1.853253309 | 5.284673513 | -2.322101873 | 0.031017118 |
| RELN | -1.85171064 | -1.582009023 | -2.621657827 | 0.016432851 |
| NUDT16L2P | 1.849691069 | 6.310854082 | 2.680647076 | 0.014458424 |
| RPL23AP30 | -1.846582196 | 0.631762149 | -2.898935707 | 0.00894076 |
| KRT8P42 | -1.846231942 | 2.122349799 | -2.612496669 | 0.016761476 |
| RNU2-22P | -1.845680133 | -1.943875294 | -3.00140414 | 0.007111147 |
| OBSL1 | -1.8433002 | 3.715966298 | -2.192555084 | 0.040468657 |
| CCDC148 | -1.839802199 | 0.286103415 | -2.143793062 | 0.044661417 |
| RN7SKP287 | -1.839793698 | -0.359932141 | -2.326949144 | 0.030706566 |
| SUGT1P1 | -1.836213903 | -1.278062093 | -2.125577797 | 0.046326314 |
| SCARNA7 | -1.829248549 | -0.934373385 | -2.502908396 | 0.021204512 |
| CIDECP2 | -1.828625253 | -1.043998313 | -2.341478256 | 0.029792859 |
| FOXD4L5 | 1.828610121 | -1.216060255 | 2.359240332 | 0.028710031 |
| ATP10B | -1.827825513 | 0.112292982 | -2.275264072 | 0.034170799 |
| LINC02763 | -1.827401421 | -0.658858984 | -2.339672517 | 0.029905033 |
| MAGIX | -1.826272928 | -1.052215129 | -2.38076605 | 0.027446673 |
| IGLV5-52 | 1.824845107 | -0.32332437 | 2.257641389 | 0.035431913 |
| MIR4782 | -1.824434203 | -1.065720113 | -2.107242814 | 0.048058763 |
| SMIM39 | -1.82322874 | -1.128305156 | -2.530977711 | 0.019971405 |
| KCTD9P4 | -1.821754213 | -1.907164539 | -2.795242357 | 0.011248542 |
| CCDC74A | -1.820460745 | 2.283145205 | -2.13670961 | 0.045302291 |
| TNRC18P3 | 1.819513535 | 0.952800892 | 2.392773691 | 0.026764557 |
| SLC25A5P5 | -1.815257633 | -1.602848438 | -2.594348964 | 0.017430876 |
| DIO2 | -1.814588149 | -1.508680488 | -2.134437344 | 0.045509636 |
| CYP1B1 | 1.807555899 | 9.00978529 | 2.900529584 | 0.008909109 |
| RNA5-8SN4 | -1.807185824 | 0.459437663 | -2.438880711 | 0.024289196 |
| SFTPA1 | -1.804951305 | -1.718145753 | -2.507541815 | 0.020996158 |
| CCND2-AS1 | -1.801057006 | 1.632290509 | -2.48982081 | 0.021803545 |
| TMEM169 | 1.80051732 | 3.452422774 | 2.468619596 | 0.022807682 |
| C2CD4D-AS1 | 1.800427305 | 0.865153574 | 2.106301767 | 0.048149243 |
| POM121L4P | -1.799795336 | 0.160212272 | -2.327177762 | 0.03069199 |
| CCR2 | 1.799336716 | 8.341754548 | 2.834937267 | 0.010304701 |
| ABALON | -1.794745136 | -1.975129596 | -3.037696794 | 0.006554322 |
| KCNJ10 | -1.793644698 | -0.245819528 | -2.137493386 | 0.045230971 |
| RPSAP6 | -1.79251373 | -1.895931719 | -2.554762916 | 0.018979547 |
| SCDP1 | -1.791601947 | -1.386331677 | -2.332311472 | 0.030366365 |
| PIK3IP1-DT | -1.79119151 | 0.746911268 | -2.09518992 | 0.049229335 |
| DPY19L1P2 | -1.790041867 | -1.896796871 | -2.625820324 | 0.016285556 |
| FAM86JP | -1.786167715 | -1.735668436 | -2.525676394 | 0.020199014 |
| ARHGEF34P | 1.78283484 | 1.244690226 | 2.243769458 | 0.036454532 |
| HMGN2P46 | -1.78172173 | -1.979687788 | -3.06166099 | 0.006210021 |
| PLK5 | -1.774702795 | -1.721214877 | -2.460786375 | 0.0231895 |
| TNFAIP8L3 | -1.774356748 | -1.738229389 | -2.476433672 | 0.022432659 |
| OCIAD1-AS1 | -1.773021376 | 0.740588125 | -2.186847328 | 0.04094018 |
| FBXO27 | -1.77185882 | -0.777576537 | -2.145820979 | 0.044479464 |
| PAQR9 | -1.767622862 | -2.213658597 | -3.061313061 | 0.006214892 |
| RNFT1P3 | -1.767616147 | -0.800291434 | -2.209341292 | 0.039110517 |
| VSIG8 | -1.76651389 | -0.736403945 | -2.093776063 | 0.049368323 |
| ADCY2 | -1.76565669 | -1.174906581 | -2.244176208 | 0.036424165 |
| THBS2-AS1 | 1.764777188 | 1.447689176 | 2.715650573 | 0.013395497 |
| FAM131B-AS1 | -1.764022943 | -2.214918568 | -3.036322802 | 0.006574612 |
| EEF2K | -1.761277219 | 3.520869314 | -2.468873859 | 0.022795387 |
| LINC02709 | -1.757324302 | 0.292441679 | -2.137917347 | 0.045192435 |
| POT1-AS1 | -1.755962014 | 0.282479055 | -2.17407002 | 0.042013938 |
| SRGAP2-AS1 | 1.753138954 | -0.794547416 | 2.160078863 | 0.043219079 |
| TNFSF18 | -1.751698152 | -1.380386675 | -2.279622352 | 0.033865345 |
| HNRNPCP4 | -1.748711839 | -1.350275159 | -2.228285602 | 0.037627903 |
| DINOL | -1.747178584 | -1.460458405 | -2.1422785 | 0.044797751 |
| PDIA3P2 | -1.745875652 | -0.42721702 | -2.158897335 | 0.043322277 |
| ATP11AUN | -1.743767889 | -1.343926395 | -2.179432787 | 0.041560183 |
| RNF5 | -1.741287061 | -1.643838965 | -2.194479222 | 0.040310822 |
| LIX1L-AS1 | 1.740972835 | 1.674541726 | 2.560804759 | 0.018735082 |
| PAX3 | -1.73832848 | -1.691105384 | -2.337382115 | 0.030047878 |
| DGCR6 | -1.738265854 | 0.105843889 | -2.315734082 | 0.031429497 |
| SNORA40B | -1.738118959 | -1.616172345 | -2.163781235 | 0.042897148 |
| NFKBIB | -1.737506564 | -1.009474364 | -2.180092127 | 0.041504705 |
| TBX10 | -1.735824403 | -0.80543267 | -2.099547878 | 0.048803151 |
| HERC2P9 | -1.733292042 | -0.811545743 | -2.135980906 | 0.045368692 |
| YAP1 | -1.732472764 | -1.668473455 | -2.266667045 | 0.034780772 |
| PLA2G10DP | -1.726423679 | -2.228078311 | -3.062664022 | 0.006195999 |
| ADORA2A-AS1 | -1.725254743 | 7.529777439 | -2.313948291 | 0.03154605 |
| USH2A | -1.724615236 | -1.721807418 | -2.377221917 | 0.027651069 |
| PRDM12 | -1.723866598 | -1.242418811 | -2.287474073 | 0.033321384 |
| FKBP2 | -1.72016738 | 1.709722715 | -2.300314836 | 0.032449101 |
| PDE4DIPP7 | 1.70896075 | -0.157166537 | 2.240662956 | 0.036687218 |
| PDK4-AS1 | 1.708245404 | 1.120926533 | 2.110988263 | 0.047700168 |
| LINC02556 | -1.708083794 | -1.373095949 | -2.12899046 | 0.046010172 |
| ZNRF2P1 | -1.707296174 | 1.653065207 | -2.184762833 | 0.041113627 |
| SDR16C5 | -1.706269733 | -0.745986564 | -2.254957023 | 0.035627722 |
| DNAH5 | -1.706096661 | -1.810432518 | -2.17744242 | 0.041728069 |
| SIRPAP1 | 1.70330544 | -0.070235275 | 2.115591842 | 0.04726273 |
| UBE2L4 | -1.702730669 | -0.38053232 | -2.447118952 | 0.023870035 |
| BIRC6-AS1 | -1.701007488 | -1.720389688 | -2.362266707 | 0.028529211 |
| FOSL1 | -1.700983758 | 8.592325526 | -2.238520401 | 0.036848495 |
| DLGAP1 | -1.700509583 | 1.423229196 | -2.431687581 | 0.024660786 |
| RNU6-188P | -1.698602644 | -1.424555072 | -2.271782652 | 0.034416616 |
| SORBS2-AS1 | -1.697213747 | -1.993043625 | -2.920604947 | 0.00851956 |
| COL4A2 | -1.695228722 | 2.53852609 | -2.197260634 | 0.04008366 |
| ZNF850 | -1.694371328 | 0.606185839 | -2.091386411 | 0.049604041 |
| FAM215A | 1.692698526 | -1.382349827 | 2.194390226 | 0.04031811 |
| KCNK5 | -1.691576986 | 5.01390582 | -2.96429851 | 0.007727566 |
| TMEM198 | -1.686758811 | 2.614249527 | -3.845957131 | 0.001024489 |
| CD276 | -1.685571664 | 0.249482641 | -2.39058415 | 0.026887752 |
| PDE7B | -1.673615994 | 2.831218177 | -2.307529263 | 0.031968304 |
| KCNH7 | 1.672270815 | 5.456090684 | 2.268127386 | 0.034676458 |
| FOSL1P1 | -1.663672925 | -1.942763777 | -2.527892794 | 0.020103559 |
| SETP20 | 1.662700071 | -0.256871589 | 2.216350151 | 0.038555874 |
| GPKOW | -1.661396993 | 3.422051872 | -2.213466417 | 0.0387832 |
| CARNMT1-AS1 | -1.658472461 | -0.863893718 | -2.145689025 | 0.044491283 |
| EPX | -1.65505899 | 2.856554793 | -2.137673424 | 0.045214603 |
| HMGN1P8 | 1.653287884 | 0.840019105 | 2.130202159 | 0.045898394 |
| ANKS1B | 1.650213837 | 2.124717977 | 2.572678011 | 0.018263278 |
| PPP2CA-DT | -1.648624448 | 2.452206196 | -2.186131038 | 0.040999706 |
| MIR5690 | 1.645931829 | 2.251932195 | 2.342704435 | 0.029716909 |
| GATA2-AS1 | -1.643599597 | 1.906951641 | -2.413493934 | 0.025624437 |
| RPS29P22 | -1.643103459 | -1.909462586 | -2.417231554 | 0.025423652 |
| SNAI1 | -1.641317635 | 5.68432268 | -2.267398953 | 0.034728455 |
| MAILR | -1.634860969 | 7.688718447 | -2.984764804 | 0.007381463 |
| KCNK9 | -1.634126527 | 0.094237783 | -2.095448359 | 0.049203967 |
| IL9R | 1.632745854 | -0.92149003 | 2.124037519 | 0.046469646 |
| MCM8-AS1 | -1.632316471 | -1.773743898 | -2.338723113 | 0.029964168 |
| TMC3 | 1.629977945 | 2.491770968 | 2.53249428 | 0.019906736 |
| CXCR6 | 1.628163559 | 4.977903671 | 3.102806198 | 0.005659322 |
| CD99 | -1.627517725 | 10.08622076 | -3.273786004 | 0.00383748 |
| CKLF | 1.626199516 | 2.929484515 | 2.195731506 | 0.040208401 |
| SNORD91B | -1.624761717 | -1.773694254 | -2.373386982 | 0.027873826 |
| TEX26-AS1 | -1.62468386 | -1.886218315 | -2.280756168 | 0.033786293 |
| MYCL | 1.622309455 | 7.085829364 | 2.679677043 | 0.014488991 |
| MTCO3P43 | 1.617734746 | -1.431076284 | 2.132277891 | 0.045707483 |
| MRC1 | 1.615876542 | 5.088023804 | 2.139803258 | 0.045021375 |
| RBMS2P1 | -1.613585504 | 0.789825749 | -2.087629218 | 0.049976711 |
| TMEM109-DT | -1.610396363 | -1.719621013 | -2.198435986 | 0.039988019 |
| NCBP2-AS1 | -1.608441344 | -0.714901619 | -2.412387711 | 0.025684146 |
| DCDC2 | -1.601341094 | -1.466024039 | -2.22427777 | 0.037937203 |
| TMEM272 | 1.601329912 | 6.617712694 | 2.351428805 | 0.029181674 |
| INSYN2A | -1.600986053 | -1.411249391 | -2.100792689 | 0.048682029 |
| ELL2P1 | -1.596906262 | 1.963189874 | -2.091659453 | 0.049577056 |
| OR52B6 | -1.595178138 | -1.74935479 | -2.260701144 | 0.035209928 |
| GSDME | 1.594744299 | 3.216046914 | 2.66870885 | 0.014838905 |
| OR6K4P | -1.592864522 | -0.981139222 | -2.127372872 | 0.046159777 |
| PCDHGA11 | -1.590250348 | 2.074743826 | -2.287470271 | 0.033321645 |
| MANSC1 | 1.588862516 | 3.847521673 | 2.183727288 | 0.041200042 |
| RBISP4 | -1.584854308 | 0.964937176 | -2.185955651 | 0.041014293 |
| A4GALT | -1.582735698 | -1.750337256 | -2.182385114 | 0.041312291 |
| OR52K1 | 1.580386818 | 2.996641752 | 2.193762436 | 0.040369553 |
| LINC01107 | -1.580349722 | -2.050167991 | -2.373458596 | 0.027869651 |
| KLK1 | -1.577498378 | 2.000398557 | -2.139538112 | 0.045045389 |
| UBE2H-DT | -1.57713207 | -1.944361048 | -2.416771686 | 0.025448277 |
| GIMAP8 | 1.573000642 | 9.01481493 | 4.072310323 | 0.000605096 |
| ARL4AP4 | -1.570259586 | 1.475834105 | -2.277059807 | 0.034044637 |
| RN7SKP16 | 1.570169508 | 2.306448916 | 2.187616918 | 0.040876312 |
| TRAV5 | 1.569474933 | 2.85278796 | 3.281875419 | 0.003767236 |
| GPR84-AS1 | -1.567172785 | 0.829517147 | -2.206049309 | 0.039373536 |
| UBL4B | -1.565264732 | -1.9522561 | -2.430889946 | 0.024702316 |
| INO80-AS1 | -1.563787198 | 1.820588059 | -2.104397334 | 0.048332823 |
| COX7A1 | -1.561975206 | 1.320068682 | -2.221856549 | 0.038125184 |
| VSNL1 | -1.558633169 | -2.001236398 | -2.633029922 | 0.016033385 |
| MLF1 | -1.552990519 | 4.061050821 | -3.51911238 | 0.00218478 |
| VPREB1 | -1.552842501 | -1.963696469 | -2.423711489 | 0.025079017 |
| MIR4521 | 1.544554969 | 2.657133691 | 2.172690533 | 0.042131387 |
| CFAP210 | -1.540610083 | 0.790087788 | -2.134685009 | 0.045486994 |
| SNORD121A | -1.540292211 | -1.326249127 | -2.119386575 | 0.046904885 |
| YWHAH-AS1 | -1.531652653 | 1.356529873 | -2.326496446 | 0.030735447 |
| HSPA6 | 1.530876795 | 11.40198804 | 2.335995154 | 0.030134684 |
| COL24A1 | -1.525198029 | 4.148808675 | -2.291359042 | 0.033055222 |
| FRG1HP | 1.524743732 | 1.701321816 | 2.332678347 | 0.030343217 |
| OR2AG2 | -1.524075606 | -2.298900136 | -3.042255416 | 0.006487435 |
| TNFRSF10C | 1.522747535 | 10.65746359 | 2.088621899 | 0.049878004 |
| MIR222HG | -1.520874361 | 7.310541913 | -3.687977183 | 0.001478356 |
| CHD5 | -1.515356628 | 3.39239015 | -3.312255919 | 0.003514483 |
| GPR22 | -1.515207865 | 1.198274611 | -2.17567746 | 0.041877458 |
| FUT4 | -1.514209826 | 8.424682473 | -4.856728824 | 9.83395E-05 |
| LRP6 | 1.510124591 | 2.938977248 | 2.093500192 | 0.049395483 |
| MPDZ | -1.507713621 | -1.860122716 | -2.380761247 | 0.027446949 |
| SNORD69 | -1.502051304 | -1.352314799 | -2.123592563 | 0.046511126 |
| NFKBIE | -1.497701766 | 10.84829931 | -2.4026538 | 0.02621515 |
| SHOC1 | 1.489481578 | 5.03490113 | 2.243935836 | 0.036442108 |
| LST1 | 1.486700376 | 3.33271918 | 2.100544308 | 0.048706175 |
| LMNA | -1.486232275 | 10.2360672 | -2.78239591 | 0.011571306 |
| SNORD13E | -1.484124247 | 2.627895962 | -2.193673899 | 0.040376813 |
| HIC1 | -1.479319967 | 8.213811344 | -2.099687151 | 0.048789586 |
| LINC02288 | 1.472619282 | 4.828090162 | 2.305661257 | 0.03209216 |
| AGAP5 | 1.471046629 | 1.303677673 | 2.10093025 | 0.04866866 |
| TLR8 | 1.469618892 | 10.80100379 | 2.297763411 | 0.032620727 |
| SUMO2P10 | -1.468932191 | -1.530889453 | -2.121888559 | 0.04667029 |
| NRIP3 | -1.468210992 | 5.097800255 | -2.122952236 | 0.046570879 |
| HIGD2B | -1.464994098 | -1.967156053 | -2.240030448 | 0.036734761 |
| NFKBID | -1.464573503 | 11.43160501 | -2.625915134 | 0.016282216 |
| LINC02520 | 1.463194505 | 5.058288825 | 3.294727712 | 0.003658212 |
| CMTM1 | 1.462931792 | 4.81803829 | 2.565577289 | 0.018544074 |
| LINC01762 | 1.462405265 | 1.952127571 | 2.195202025 | 0.040251677 |
| SPRY2 | -1.459555846 | 6.868057868 | -2.142063575 | 0.044817128 |
| GPR84 | -1.458570759 | 6.752082108 | -2.436185791 | 0.024427797 |
| IBA57-DT | -1.455323419 | 2.778301744 | -2.114151936 | 0.04739916 |
| DUSP10 | -1.455037796 | 8.87115017 | -2.502816255 | 0.021208675 |
| SETP9 | 1.45428762 | 1.377685795 | 2.644650574 | 0.015634689 |
| CD248 | 1.452073562 | 3.698992575 | 3.174843718 | 0.004807185 |
| TLR5 | 1.451102249 | 8.847373715 | 2.271426124 | 0.034441882 |
| JUN | -1.44973094 | 13.26545034 | -2.171689246 | 0.042216823 |
| NLRP6 | 1.448998926 | 10.79605852 | 2.402741713 | 0.026210309 |
| TLR10 | 1.444308334 | 7.990265968 | 2.495992306 | 0.02151912 |
| KIAA0040 | 1.443635696 | 11.23353196 | 2.674766977 | 0.014644656 |
| PSME2P4 | 1.441722052 | 1.70077261 | 2.319770784 | 0.031167496 |
| GIMAP4 | 1.439285404 | 11.27097662 | 2.907013687 | 0.008781454 |
| SNORA66 | -1.434474581 | 1.84897409 | -2.541070233 | 0.019544733 |
| KBTBD7 | 1.432485835 | 7.520546516 | 2.816202945 | 0.010740305 |
| SEPTIN7P10 | -1.431855135 | 1.927745242 | -2.484824703 | 0.022036374 |
| CHILL1 | 1.427306262 | 6.19385208 | 2.30511617 | 0.032128385 |
| LHFPL6 | -1.418305975 | 2.989272927 | -2.38451092 | 0.027232226 |
| P2RY13 | 1.41562211 | 10.54872447 | 2.184796082 | 0.041110855 |
| NOP2 | -1.410095981 | 4.598058295 | -3.549724109 | 0.002035723 |
| INE1 | -1.40940788 | 2.764885878 | -2.090361047 | 0.049705496 |
| MYL11 | -1.402339604 | 2.333861033 | -2.093400523 | 0.049405299 |
| SETSIP | -1.394464725 | 1.080848924 | -2.09517174 | 0.04923112 |
| TMEM132A | -1.392881945 | 4.675394872 | -2.300315978 | 0.032449024 |
| RERE-AS1 | 1.391128542 | 3.886203091 | 2.925749419 | 0.008422403 |
| RALGDS | -1.387246798 | 7.246071108 | -4.228524167 | 0.000420698 |
| IRF2BP2 | -1.382632342 | 12.18885851 | -5.415850932 | 2.76008E-05 |
| ZC3H12A | -1.382366389 | 12.56497239 | -2.243602586 | 0.036466996 |
| GAS2L3 | -1.363402265 | 4.595404012 | -2.658395288 | 0.015175233 |
| PTX3 | -1.361080257 | 7.723145877 | -3.009536851 | 0.006982529 |
| SOCS3 | 1.354519545 | 12.05817084 | 2.548325295 | 0.019243327 |
| IGSF6 | 1.351309629 | 8.687233281 | 2.09217002 | 0.049526633 |
| POLR1F | -1.349654616 | 7.598615125 | -2.374284598 | 0.027821538 |
| TRAV9-2 | 1.349314392 | 2.810386972 | 2.7517234 | 0.012378075 |
| SPDYE16 | 1.344977558 | 2.714588302 | 3.713179201 | 0.001394445 |
| OLFM2 | 1.342774033 | 3.958727401 | 2.80938929 | 0.010903059 |
| GNA15-DT | -1.342728406 | 4.054066672 | -4.151482476 | 0.000503278 |
| HMGCS1 | -1.33217442 | 9.2764224 | -3.807193543 | 0.001121051 |
| VSIG4 | 1.329045866 | 7.302445671 | 2.110929555 | 0.04770577 |
| SLC6A12 | 1.326107245 | 5.661962679 | 2.190506647 | 0.040637307 |
| IRGQ | -1.316916376 | 9.297952369 | -3.191149552 | 0.00463238 |
| SLC22A1 | 1.307426461 | 6.634425888 | 2.390904742 | 0.026869681 |
| KCTD12 | 1.306779394 | 11.1679132 | 2.742768894 | 0.012623538 |
| ZKSCAN7 | 1.305478353 | 2.751439994 | 2.641698125 | 0.015735086 |
| DOCK6 | -1.300807467 | 5.105881379 | -2.823980745 | 0.01055736 |
| CD8B2 | 1.294160796 | 2.93554708 | 2.389654769 | 0.026940203 |
| SLC25A33 | -1.293075006 | 7.359805327 | -3.799890367 | 0.001140232 |
| PRKAR2A-AS1 | 1.290468844 | 2.189855045 | 2.099557978 | 0.048802167 |
| ANKRD34B | 1.284208003 | 6.342018882 | 2.381770483 | 0.027389002 |
| MTCYBP11 | 1.282092412 | 1.590360676 | 2.244985261 | 0.036363833 |
| TNFSF10 | 1.277557417 | 10.32255924 | 2.598515976 | 0.017274976 |
| RPL34P22 | -1.277028072 | 2.899314972 | -3.141400208 | 0.005186025 |
| CALB1 | 1.272507151 | 3.361991118 | 3.231054145 | 0.004230297 |
| NR1I3 | -1.26780353 | 4.463695223 | -3.569963915 | 0.001942725 |
| GTSF1 | 1.265634809 | 4.725147226 | 4.134962406 | 0.000523002 |
| MAPK6 | -1.264655044 | 9.44396106 | -4.125084701 | 0.000535163 |
| C9orf64 | 1.26129838 | 5.190891835 | 3.499642317 | 0.00228513 |
| WDFY3-AS2 | 1.260699692 | 3.088149974 | 2.09582074 | 0.049167437 |
| TMEM269 | -1.256445852 | 2.594213015 | -2.342307403 | 0.029741482 |
| FAM171A1 | 1.256305267 | 4.410697114 | 3.470406496 | 0.002444396 |
| CRY1 | -1.256118358 | 8.875277781 | -4.359656872 | 0.000310171 |
| TTC9 | 1.253465934 | 8.364263291 | 2.668480045 | 0.014846289 |
| SORT1 | 1.247978893 | 10.36454277 | 2.178224293 | 0.041662045 |
| NTRK1 | -1.246151601 | 5.42172828 | -2.593380844 | 0.017467286 |
| ARL2BP | -1.240607102 | 2.966325558 | -2.910693941 | 0.008709784 |
| TLR1 | 1.236410312 | 11.09285578 | 2.420914739 | 0.025227225 |
| FTH1P22 | 1.235959421 | 4.668960118 | 2.599810191 | 0.017226824 |
| CRIP2 | -1.235858412 | 8.11808474 | -2.111739402 | 0.047628544 |
| CEBPD | 1.223112094 | 12.67011156 | 2.407600073 | 0.025944058 |
| TRAV4 | 1.22283334 | 3.891082459 | 2.309106346 | 0.03186408 |
| TRAF4 | -1.220765324 | 8.926814001 | -2.830430083 | 0.010407929 |
| PTK7 | -1.219467381 | 5.651679581 | -2.102510225 | 0.048515356 |
| NLRC4 | 1.21664909 | 6.149843964 | 2.464538396 | 0.023005874 |
| AIM2 | 1.215428389 | 7.673375757 | 2.125288749 | 0.046353181 |
| DHRS13 | 1.21222084 | 8.049932539 | 2.35640514 | 0.028880391 |
| FZD3 | -1.211940437 | 5.777515475 | -2.36417331 | 0.028415838 |
| B3GALNT2 | -1.208782023 | 3.001612653 | -2.354274006 | 0.029009062 |
| CCR5 | 1.198387337 | 6.81347847 | 2.358799879 | 0.028736435 |
| ARL5B | -1.195150549 | 10.97552294 | -2.569110455 | 0.018403851 |
| ZDHHC1 | -1.191528047 | 5.505516526 | -2.61831898 | 0.016551909 |
| KRT18P34 | 1.191228593 | 2.979981035 | 2.688787568 | 0.014204307 |
| FAM110C | 1.190488241 | 3.515302225 | 2.578898232 | 0.018020597 |
| GIMAP7 | 1.189872208 | 8.489641436 | 3.20991975 | 0.004438814 |
| MEX3B | -1.186144473 | 5.212078766 | -2.251860457 | 0.035854832 |
| PIANP | 1.185746295 | 3.560902174 | 3.956095633 | 0.000793 |
| ID1 | -1.184590738 | 7.317431099 | -2.998038212 | 0.007165046 |
| SLC35B2 | -1.184139141 | 9.930104477 | -3.54100129 | 0.002077144 |
| MAD2L1-DT | 1.184069657 | 1.122864034 | 2.535897052 | 0.019762353 |
| MIR503HG | 1.180893276 | 3.086127865 | 2.902160555 | 0.008876832 |
| TSPY26P | -1.180308391 | 6.682652668 | -4.655814174 | 0.000156193 |
| PEAK3 | 1.179149551 | 8.812121028 | 2.385236507 | 0.027190856 |
| LINC01465 | -1.178347433 | 3.866434443 | -2.519993091 | 0.020445727 |
| DHRS9 | 1.177743899 | 8.182991113 | 2.161145196 | 0.043126134 |
| ZNF366 | 1.1753035 | 3.490050139 | 2.368672938 | 0.028149929 |
| LZTS3 | -1.172797939 | 6.903598457 | -2.200751095 | 0.039800242 |
| TUBBP1 | -1.168971499 | 4.069164144 | -2.307495223 | 0.031970557 |
| SLCO4A1-AS2 | 1.166850134 | 5.325501824 | 2.618142939 | 0.016558209 |
| YES1 | -1.163326746 | 7.118741356 | -2.770964065 | 0.011865947 |
| TNFAIP8L2 | 1.160456873 | 8.249599617 | 2.946858182 | 0.008034796 |
| LINC02432 | 1.158601871 | 5.103822829 | 2.291423756 | 0.033050805 |
| SKOR1 | -1.157269653 | 4.94765659 | -2.687591684 | 0.01424137 |
| CLDN9 | 1.156486934 | 6.86228159 | 2.116295616 | 0.047196178 |
| EFCAB8 | 1.154790696 | 4.515078378 | 2.950005758 | 0.007978492 |
| SIRPG | 1.154025577 | 7.055339324 | 3.124105921 | 0.005393158 |
| PIWIL4 | -1.151473544 | 6.990790221 | -3.614682139 | 0.001751868 |
| PLTP | -1.151368052 | 4.905203068 | -2.458099259 | 0.02332185 |
| HIVEP2-DT | -1.147839199 | 3.141425326 | -2.527112072 | 0.020137134 |
| P2RY2 | -1.143959763 | 7.4028607 | -2.331232 | 0.030434568 |
| ZBTB10 | -1.140679499 | 8.743944532 | -2.436710516 | 0.024400752 |
| SH3D19 | -1.140357857 | 3.38902407 | -3.028672065 | 0.006688712 |
| UPB1 | -1.136218342 | 5.849566231 | -2.610759347 | 0.016824493 |
| KBTBD6 | 1.135727798 | 5.5408042 | 2.460046066 | 0.023225893 |
| TMEM121B | 1.134454897 | 8.366550109 | 2.529330327 | 0.020041875 |
| FAM183BP | -1.132651367 | 4.007497926 | -2.28916969 | 0.033204974 |
| LINC02986 | -1.130579412 | 3.35276238 | -2.863017168 | 0.009683339 |
| TRAV21 | 1.130434983 | 3.086993454 | 2.309790002 | 0.031818997 |
| SIGLEC9 | 1.128887075 | 9.630136121 | 2.12390562 | 0.046481938 |
| MFSD2A | -1.127453442 | 7.038477534 | -2.327564046 | 0.030667377 |
| PTPRS | -1.126750233 | 6.239107091 | -2.236141727 | 0.037028306 |
| CHAC1 | -1.125620965 | 2.843984709 | -2.204167279 | 0.039524629 |
| RPS4XP1 | 1.125405703 | 2.997811619 | 3.20617431 | 0.004476798 |
| SLC7A5 | -1.124479246 | 10.23132547 | -2.131042498 | 0.045821019 |
| AATBC | 1.124062358 | 6.660197803 | 2.519701533 | 0.02045846 |
| NKX3-1 | -1.12238326 | 5.308943288 | -2.746303684 | 0.012526092 |
| ARHGEF39 | -1.121749551 | 3.407890646 | -2.874303231 | 0.009443858 |
| LINC02705 | 1.111985013 | 4.345085954 | 2.12908618 | 0.046001333 |
| OR52B3P | 1.111153964 | 1.426416599 | 2.710254634 | 0.013554364 |
| CDH26 | 1.107588857 | 5.891874295 | 2.353962231 | 0.02902793 |
| PPP1R1A | -1.107334836 | 2.229245344 | -2.578120564 | 0.01805077 |
| PA2G4P6 | 1.1072837 | 3.376786791 | 3.646716751 | 0.001626658 |
| AVIL | 1.10143557 | 8.067943448 | 2.334679401 | 0.030217249 |
| TRAIP | -1.101231837 | 5.240356128 | -2.314116034 | 0.031535085 |
| SGK1 | -1.097587647 | 12.51906936 | -2.213705549 | 0.038764302 |
| ATP2B1 | -1.09411088 | 11.94749988 | -4.923124805 | 8.44489E-05 |
| CARD6 | 1.087155625 | 7.975033674 | 2.30052093 | 0.032435274 |
| ELL2 | -1.083029708 | 10.22296454 | -2.788599362 | 0.011414357 |
| TRAV17 | 1.082112306 | 3.592265258 | 2.107882764 | 0.047997321 |
| MIR133A1HG | -1.080684798 | 1.458632317 | -3.02855761 | 0.006690433 |
| RASL10A | -1.080443911 | 2.260504574 | -2.479467376 | 0.022288622 |
| CR2 | 1.080070327 | 5.697006081 | 2.490085788 | 0.021791261 |
| IFITM10 | -1.076614365 | 3.917445895 | -2.35668467 | 0.028863553 |
| NRBF2 | 1.076351013 | 10.9864359 | 2.229274861 | 0.037551914 |
| RNF157-AS1 | 1.075044424 | 3.954103545 | 2.424830896 | 0.025019924 |
| STX18-AS1 | -1.07425159 | 2.830178776 | -3.230849651 | 0.004232268 |
| MATCAP2 | -1.072769892 | 4.128545609 | -2.313735145 | 0.031559988 |
| PNP | -1.071070123 | 4.911012383 | -3.177824061 | 0.004774762 |
| RNU6-892P | 1.070834564 | 2.67911414 | 2.834914435 | 0.010305221 |
| PPP1R3B | 1.068836791 | 7.805013475 | 2.229458141 | 0.037537851 |
| ODAD4 | -1.064414923 | 3.428920093 | -2.451721303 | 0.023638817 |
| ENPP2 | -1.062646581 | 4.78726923 | -2.306455392 | 0.032039452 |
| SLC25A29 | -1.059174879 | 8.906106442 | -2.523323763 | 0.020300801 |
| SEMA4B | 1.058056515 | 11.16365076 | 2.418743886 | 0.025342826 |
| CACNB3 | -1.054628189 | 6.766631347 | -2.902699296 | 0.008866196 |
| DIABLO | -1.05338364 | 5.266628798 | -2.174285626 | 0.041995608 |
| PRSS8 | 1.05291313 | 2.196189307 | 2.405971548 | 0.026033023 |
| PDE4DIPP6 | 1.052308207 | 2.902569276 | 3.018795431 | 0.006838841 |
| DHRS1 | -1.049927595 | 4.479832775 | -2.509728193 | 0.02089851 |
| HKDC1 | 1.049680952 | 4.58921769 | 3.05990455 | 0.00623465 |
| SAXO2 | -1.048660473 | 2.320237641 | -2.093449779 | 0.049400448 |
| SLC4A11 | -1.046279377 | 3.184533417 | -2.358830388 | 0.028734605 |
| ACMSD | 1.046218207 | 1.342562211 | 2.196946868 | 0.040109227 |
| OTULINL | 1.046020578 | 9.742177504 | 2.45251375 | 0.023599217 |
| OPRL1 | 1.044099645 | 4.735096501 | 2.573381941 | 0.01823566 |
| CLCN4 | 1.04207468 | 7.6290793 | 2.128150757 | 0.046087779 |
| DPY19L1P1 | -1.037121153 | 3.368057684 | -2.166619592 | 0.042651825 |
| NFASC | -1.031647253 | 2.892535072 | -2.335416614 | 0.030170962 |
| PLK2 | -1.031347024 | 7.852763324 | -2.369713878 | 0.028088743 |
| EIF2AK3 | -1.030449181 | 8.91748886 | -3.05232223 | 0.00634206 |
| AOC3 | 1.030250971 | 7.651335833 | 2.22438808 | 0.037928659 |
| ABCC6 | 1.029344858 | 3.554573695 | 2.311483812 | 0.031707555 |
| TP73 | -1.029313483 | 4.150969888 | -3.178541658 | 0.004766987 |
| PKD2L2-DT | 1.02627805 | 1.924454362 | 2.517122547 | 0.02057141 |
| ZFP3 | 1.025165787 | 5.001800419 | 3.971416371 | 0.000765231 |
| SYNRG | 1.021479153 | 5.837758431 | 2.768483959 | 0.011930807 |
| CHKA | -1.021354683 | 7.877149919 | -3.967401133 | 0.000772413 |
| PPP1CB-DT | 1.019800184 | 2.550741821 | 2.398549955 | 0.026442068 |
| TMEM229B | 1.018951658 | 7.842494857 | 2.958711217 | 0.007824739 |
| SOWAHD | 1.017033209 | 6.046340457 | 2.400913709 | 0.026311145 |
| EBAG9P1 | 1.015464375 | 2.549404051 | 2.712757362 | 0.013480457 |
| SLC35E4 | -1.011854757 | 5.355093649 | -2.400906401 | 0.026311548 |
| GALNT5 | -1.010944321 | 3.16093049 | -2.4678595 | 0.022844472 |
| CXCL3 | -1.010932663 | 5.000247275 | -2.106879169 | 0.048093709 |
| ST20 | 1.008411523 | 4.655209535 | 2.271330366 | 0.03444867 |
| RESF1 | 1.005687254 | 13.30498959 | 2.309153083 | 0.031860996 |
| BNIP1 | -1.004626497 | 6.818891141 | -2.365567572 | 0.028333195 |
| PRMT5-DT | 1.003486919 | 5.169015172 | 2.182298483 | 0.041319546 |
| ADAM11 | -1.00163103 | 3.825787392 | -2.914316552 | 0.008639786 |
